# Energy Dissipation and Decoherence in Solid-State Quantum Devices: Markovian versus non-Markovian Treatments

**DOI:** 10.3390/e22040489

**Published:** 2020-04-24

**Authors:** Rita Claudia Iotti, Fausto Rossi

**Affiliations:** Department of Applied Science and Technology, Politecnico di Torino, Corso Duca degli Abruzzi 24, 10129 Torino, Italy; fausto.rossi@polito.it

**Keywords:** semiconductor nanodevices, electronic phase coherence, dissipation models, Markov limit, quantum technologies, density-matrix formalism

## Abstract

The design and optimization of new-generation solid-state quantum hardware absolutely requires reliable dissipation versus decoherence models. Depending on the device operational condition, the latter may range from Markov-type schemes (both phenomenological- and microscopic- like) to quantum-kinetic approaches. The primary goal of this paper is to review in a cohesive way virtues versus limitations of the most popular approaches, focussing on a few critical issues recently pointed out (see, e.g., Phys. Rev. B **90**, 125140 (2014); Eur. Phys. J. B **90**, 250 (2017)) and linking them within a common framework. By means of properly designed simulated experiments of a prototypical quantum-dot nanostructure (described via a two-level electronic system coupled to a phonon bath), we shall show that both conventional (i.e., non-Lindblad) Markov models and density-matrix-based non-Markov approaches (i.e., quantum-kinetic treatments) may lead to significant positivity violations. While for the former case the problem is easily avoidable by choosing genuine Lindblad-type dissipation models, for the latter, a general strategy is still missing.

## 1. Introduction

Quantum-mechanical state superposition and correlation (i.e., entanglement) are the key players for the concrete realization of quantum information processing devices [1,2]. In particular, the pivotal ingredient for many solid-state implementations is electronic phase coherence [3,4]. The latter is, however, strongly hindered by dissipation and/or decoherence phenomena [5]. For the design and optimization of new-generation electronic quantum devices [6,7], it is then imperative to employ reliable dissipation versus decoherence models. The most popular quantum-mechanical picture for the description of phase coherence versus dissipation/decoherence in open quantum systems is the well-known density-matrix formalism [8], recalled and applied to the case of semiconductor nanodevices in Section 2. Within this picture, the simplest example is a two-level system, described by the following (two-by-two) density matrix:

(1) $$\left(\begin{array}{cc} \rho_{bb} & \rho_{ba}\\ \rho_{ab} & \rho_{aa} \end{array}\right)=\left(\begin{array}{cc} f_{b} & p\\ p^{*} & f_{a} \end{array}\right)$$

in terms of the ground- and excited-level populations, $f_{a}$ and $f_{b}$ respectively, as well as of the interlevel phase coherence (or polarization) *p*. In this case, the simplest dissipation model is the well-known $T_{1}/T_{2}$ scheme [7]. This fully phenomenological approach accounts for dissipation versus decoherence processes via bare relaxation-time approximations: the evolution of the density matrix in Equation (1) is described by the following set of coupled equations:

(2) $$\frac{df_{a/b}}{dt}=-\frac{f_{a/b}-f_{a/b}^{{}^{\circ}}}{T_{1}}\;,\;\frac{dp}{dt}=\frac{\Delta_{c}}{ı\hbar }\;p\;-\;\frac{p}{T_{2}}$$

($\Delta_{c}=\epsilon_{b}-\epsilon_{a}$ denoting the interlevel energy splitting), for which the solution is simply given by

(3) $$f_{a/b}\left(t\right)=f_{a/b}^{{}^{\circ}}+\left(f_{a/b}\left(0\right)\right)e^{-\frac{t}{T_{1}}}\;,\;p\left(t\right)=p\left(0\right)e^{\frac{\Delta_{c}t}{ı\hbar }}e^{-\frac{t}{T_{2}}}\;.$$

At long times, the level populations $f_{a/b}$ tend to their thermal-equilibrium values $f_{a/b}^{{}^{\circ}}$ with a relaxation time $T_{1}$, while the interlevel polarization *p* decays to zero with another relaxation time, $T_{2}$. In spite of its success in the interpretation of many ultrafast optical experiments [9], the $T_{1}/T_{2}$ model may lead to totally unphysical results when the two relaxation times are treated as independent parameters.

**Figure 1:**
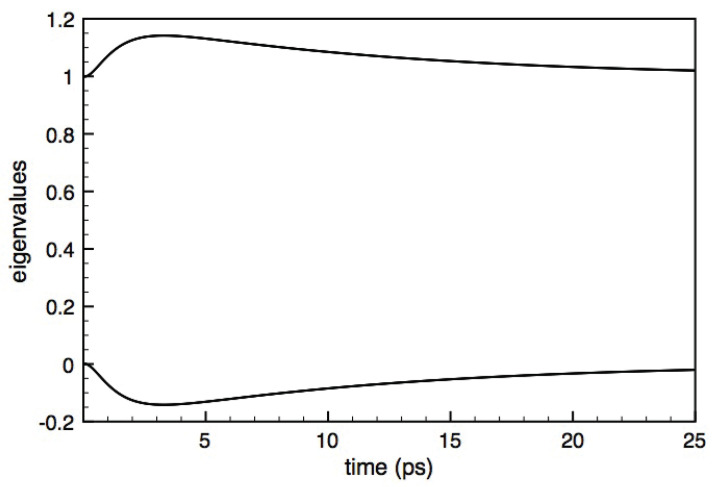
Time evolution of the eigenvalues $\Lambda_{\pm }$ of the density matrix in Equation (1) corresponding to a two-level system treated within the $T_{1}/T_{2}$ model in Equation (3) in the low-temperature limit, that is, $f_{a}^{{}^{\circ}}=1,f_{b}^{{}^{\circ}}=0$: Here, the system is initially prepared in a so-called Bell state, $f_{a}\left(0\right)=f_{b}\left(0\right)=p\left(0\right)=\frac{1}{2}$, and the two relaxation times are $T_{1}=1$ ps and $T_{2}=20$ ps. For this particular parameter choice, the lowest eigenvalue becomes negative, which implies that the density matrix becomes not positive-definite.

Indeed, as discussed below, a crucial prerequisite of any reliable dissipation model is to preserve the positivity of the density matrix, namely the positive-definite character of its eigenvalues. For the case of our two-level system, such basic requirement translates into the condition that the eigenvalues

(4) $$\Lambda_{\pm }=\frac{1}{2}\left(\left(f_{a}+f_{b}\right)\pm \sqrt{{\left(f_{a}-f_{b}\right)}^{2}+4{\left|p\right|}^{2}}\right)$$

of the density matrix in Equation (1) are nonnegative or, equivalently, that the determinant $f_{a}f_{b}-{\left|p\right|}^{2}$ is nonnegative. Positivity violation therefore does not necessarily correspond to a negative value of either $f_{a}$ or $f_{b}$, as it may also occur in the presence of nonnegative level populations. This is the case, for example, of the $T_{1}/T_{2}$ solution in Equation (3) for particular combinations of the two phenomenological parameters, as shown in Figure 1. This pathological behavior is well known and is ascribed to the fact that the two relaxation times $T_{1}$ and $T_{2}$ are not physically independent quantities; indeed, by adopting more refined descriptions [7], they may be expressed in terms of microscopic scattering rates corresponding to the various interaction processes. The first obvious conclusion drawn from the above scenario is the potential inadequacy of barely phenomenological models and therefore the need for fully microscopic treatments.

The microscopic derivation of reliable scattering superoperators is one of the most challenging problems in quantum physics. For purely atomic and/or photonic systems, dissipation and decoherence phenomena may successfully be treated via adiabatic-decoupling schemes [8] in terms of extremely simplified models based on a few key parameters; within such effective treatments, the main goal is to derive a suitable form of the system-environment Liouville superoperator, able to preserve the positivity of the system density matrix [10]. This is typically accomplished by identifying so-called Lindblad superoperators [11] expressed in terms of a few crucial system–environment coupling constants. In contrast, for solid-state materials and related devices, the complex many-body quantum evolution results in a highly nontrivial interplay between electronic phase coherence and dissipation/decoherence [12,13,14,15,16], thus requiring the adoption of microscopic treatments.

Generally speaking, the microscopic derivation of solid-state dissipation models within the density-matrix picture may involve one or more of the following three key steps: (i) mean-field approximation, (ii) adiabatic or Markov limit, and (iii) semiclassical or diagonal approximation.

When all these three steps/approximations are performed, the collision term familiar from the Boltzmann theory is obtained; the latter, when applicable, constitutes a robust/reliable particle-like description in purely stochastic terms, thus providing in any case physically acceptable results.

In contrast, the combination of the first two steps only, namely mean-field approximation and adiabatic limit, allows one to derive so-called Markovian scattering superoperators, for which action may lead again to positivity violations [17]. Indeed, as originally pointed out by Spohn and coworkers [18], the choice of the adiabatic decoupling strategy is definitely not unique. Only the case studied by Davies [10], namely a “small” subsystem interacting with a thermal environment, could be shown to preserve positivity. However, such result was restricted to finite-dimensional subsystems (e.g., few-level atoms) and to the particular projection scheme of the partial trace. Thus, as such, it cannot be straightforwardly extended to the study of solid-state systems.

To overcome this severe limitation, a few years ago, an alternative and more general Markov procedure has been proposed [19]; the latter allows for a microscopic derivation of Lindblad-type scattering superoperators [11], thus preserving the positive-definite nature of the electronic density matrix. More recently, such an alternative Markov scheme combined with the conventional mean-field approximation has allowed for the derivation of a positive-definite nonlinear equation for the single-particle density matrix [20,21], able to describe both carrier–phonon and carrier–carrier interaction; the latter has been recently applied to the investigation of scattering nonlocality in GaN-based materials [22] and carbon nanotubes [23] as well as to the study of carrier capture processes [24,25].

For strong (i.e., non-perturbative) system–environment couplings combined with extremely short excitation and/or detection timescales, the adoption of the adiabatic or Markov schemes just recalled becomes questionable, and memory effects may be investigated within the density-matrix formalism via so-called quantum-kinetic schemes [12,15]. Indeed, stimulated by the pioneering papers by Haug and coworkers [26] as well as by Kuhn and coworkers [27], over the last decade, several groups have routinely employed such non-Markovian techniques to study a wide spectrum of ultrafast coherent phenomena in semiconductor bulk and nanostructures [28,29,30,31,32,33,34,35,36,37,38,39,40,41,42,43,44,45,46,47,48,49,50,51,52,53,54,55,56,57,58,59,60,61]. In spite of the undoubted success, these quantum-kinetic treatments, based on the mean-field approximation only, may lead once again to positivity violations. Indeed, such potential limitation, originally pointed out in the early days of electron-phonon quantum kinetics by Zimmermann and coworkers [62], has been recently investigated in more detail [63,64].

The primary goal of this paper is to review in a cohesive way virtues versus limitations of the most used dissipation quantum models employed in the simulation of state-of-the-art electronic and optoelectronic nanodevices, focussing on a few critical issues recently pointed out in Reference [21] as well as in Reference [64] and linking them within a common framework. More specifically, by means of properly designed simulated experiments of a prototypical quantum-dot nanostructure (described via an electronic two-level system coupled to a phonon bath), we shall show the following:

(i) conventional (i.e., non-Lindblad) Markov models may lead to significant positivity violations;
(ii) such intrinsic limitations may be avoided adopting properly designed Lindblad-type Markov schemes;
(iii) density-matrix-based non-Markov models, namely quantum-kinetic treatments, may lead to positivity violations as well.

The most important conclusion of our investigation is that the presence of positivity violations is ascribed not only to the adiabatic approximation but also to the mean-field approximation. Indeed, while for the case of Markov treatments (based on both adiabatic and mean-field approximations), the problem is easily avoidable by employing genuine Lindblad-type dissipation models, for the case of non-Markov treatments (based on the mean-field approximation only), a general strategy is still missing. As a result, the unusual conclusion is that, in this case, two approximations work better than one.

The paper is organized as follows: In Section 2, we shall focus on Markovian models, comparing the conventional adiabatic-decoupling scheme with the Lindblad-type one and generalizing the latter to the nonlinear (i.e., degenerate) regime. In Section 3, we shall discuss virtues versus limitations of density-matrix-based non-Markovian models, namely quantum-kinetic treatments, pointing out specific conditions/regimes which may lead to positivity violations. Finally, in Section 4, we shall summarize and draw a few conclusions.

## 2. Markovian Dissipation Models

Within the spirit of the usual perturbation theory, the global solid-state Hamiltonian (electrons plus various crystal excitations, e.g., phonons, plasmons, etc.) may be schematically written as

(5) $$\hat{H}={\hat{H}}_{{}^{\circ}}+\sum_{s}{\hat{H}}_{s}^{\prime }\;,$$

where the first term is the unperturbed contribution that can be treated exactly and the second term describes a number of perturbations ${\hat{H}}_{s}^{\prime }$, corresponding to various interaction mechanisms (e.g., carrier–phonon, carrier–carrier, etc.), which are typically treated within some approximation scheme.

### 2.1. Conventional Adiabatic-Decoupling Scheme

Following the fully operatorial approach originally proposed in Reference [17]) and described in more detail in Reference [7], the second-order (or incoherent) contribution to the time evolution of the global (e.g., carriers plus phonons) density-matrix operator $\hat{\rho }$ obtained via the conventional adiabatic limit can be written as

(6) $${\left.\frac{d\hat{\rho }}{dt}\right|}_{\mathrm{inco}}=\frac{1}{2}\sum_{s}\left({\hat{a}}^{s}\hat{\rho }{\hat{b}}^{s†}-{\hat{a}}^{s†}{\hat{b}}^{s}\hat{\rho }\right)\;+\;H.c.\;,$$

where

(7) $${\hat{a}}^{s}=\frac{{\hat{H}}_{s}^{\prime }}{\hbar }\;,\;{\hat{b}}^{s}=\frac{1}{\hbar }\int_{-\infty }^{+\infty }e^{-\frac{{\hat{H}}_{{}^{\circ}}t^{\prime }}{ı\hbar }}{\hat{H}}_{s}^{\prime }e^{\frac{{\hat{H}}_{{}^{\circ}}t^{\prime }}{ı\hbar }}dt^{\prime }\;,$$

and H.c. denotes the Hermitian conjugate.

The above Markov evolution is definitely non-Lindblad and therefore does not necessarily preserve the positivity of the global density matrix $\hat{\rho }$ [19]. The scattering superoperator in Equation (6) may suitably be expressed in terms of generalized scattering rates. More specifically, denoting with |i〉 the generic eigenstate of the noninteracting Hamiltonian ${\hat{H}}_{{}^{\circ}}$ and with $\epsilon_{i}$ the corresponding energy level, one gets

(8) $${\left.\frac{d\rho_{i_{1}i_{2}}}{dt}\right|}_{\mathrm{inco}}=\frac{1}{2}\;\sum_{s,i_{1}^{\prime }i_{2}^{\prime }}\left(P_{i_{1}i_{2},i_{1}^{\prime }i_{2}^{\prime }}^{s}\rho_{i_{1}^{\prime }i_{2}^{\prime }}-P_{i_{1}^{\prime }i_{1}^{\prime },i_{1}i_{2}^{\prime }}^{s*}\rho_{i_{2}^{\prime }i_{2}}\right)+H.c.$$

with generalized scattering rates

(9) $$P_{i_{1}i_{2},i_{1}^{\prime }i_{2}^{\prime }}^{s}=a_{i_{1}i_{1}^{\prime }}^{s}b_{i_{2}i_{2}^{\prime }}^{s*}\;.$$

It is possible to show [7] that their diagonal (i.e., semiclassical) elements ($i_{1}i_{1}^{\prime }=i_{2}i_{2}^{\prime }$) coincide with the standard Fermi’s-golden-rule prescription:

(10) $$P_{ii,i^{\prime }i^{\prime }}^{s}=\frac{2\pi }{\hbar }{\left|\langle i|{\hat{H}}_{s}^{\prime }|i^{\prime }\rangle \right|}^{2}\delta \left(\epsilon_{i}-\epsilon_{i^{\prime }}\right)\;.$$

Since the study of electro-optical processes in solid-state systems mainly relies on physical quantities that depend on the electronic-subsystem coordinates only, it is customary to introduce a many-body density-matrix operator

(11) $${\hat{\rho }}_{c}=\mathrm{tr}{\left\{\hat{\rho }\right\}}_{p}\;,$$

where the nonrelevant phononic (p) degrees of freedom have been traced out of the global density-matrix operator $\hat{\rho }$. It is worth stressing that such treatment of carrier–phonon interaction applies to other bosonic degrees of freedom as well (e.g., photons, plasmons, etc.).

More specifically, by denoting with ${\hat{\rho }}_{p}^{{}^{\circ}}$ the equilibrium density-matrix operator of the phononic subsystem and by adopting a carrier–phonon mean-field approximation via the following state factorization

(12) $$\hat{\rho }={\hat{\rho }}_{c}⊗{\hat{\rho }}_{p}^{{}^{\circ}}\;,$$

it is possible to show [7] that the reduced dynamics dictated by the global evolution in Equation (6) is still of the same form:

(13) $${\left.\frac{d{\hat{\rho }}_{c}}{dt}\right|}_{\mathrm{inco}}=\frac{1}{2}\sum_{s}\left({\hat{a}}_{c}^{s}{\hat{\rho }}_{c}{\hat{b}}_{c}^{s†}-{\hat{a}}_{c}^{s†}{\hat{b}}_{c}^{s}{\hat{\rho }}_{c}\right)\;+\;H.c.\;.$$

Here, the explicit form of the reduced or electronic operators ${\hat{a}}_{c}^{s}$ and ${\hat{b}}_{c}^{s}$ can be derived starting from the global scattering operators ${\hat{a}}^{s}$ and ${\hat{b}}^{s}$ in Equation (7). We stress that, in spite of their very same formal structure, Equations (6) and (13) describe the system dynamics at different levels. This is confirmed by the fact that, while the global operators in Equation (7) are always Hermitian, the electronic ones, ${\hat{a}}_{c}^{s}$ and ${\hat{b}}_{c}^{s}$, are generally non-Hermitian, a clear fingerprint of dissipation-versus-decoherence processes induced by the phononic subsystem on the carrier one.

Within the above description, although a statistical average over the phononic degrees of freedom has been performed, the electronic subsystem is still treated via a many-body picture. Nevertheless, in the investigation of solid-state quantum materials and related devices, many of the physical quantities of interest are described via single-particle electronic operators. This suggests to treat the electronic subsystem via an additional mean-field (or Hartree–Fock) approximation. When applicable [7], this last approximation step allows one to get nonlinear single-particle dissipation models (see Section 2.3). In the low-density limit/regime, however, carrier–carrier interaction as well as Pauli-blocking effects can safely be neglected, and the many-body Hartree–Fock scheme just mentioned can conveniently be replaced by a simple one-electron model, i.e., the properties of a system of noninteracting electrons are fully described by one electron only. This amounts to treating the carrier subsystem in terms of a one-electron density matrix $\rho_{\alpha_{1}\alpha_{2}}$, i.e.,

(14) $${\hat{\rho }}_{c}=\sum_{\alpha_{1}\alpha_{2}}|\alpha_{1}\rangle \rho_{\alpha_{1}\alpha_{2}}\langle \alpha_{2}|\;,$$

where |α〉 denotes the generic one-electron eigenstate.

In the particular yet physically relevant case of a low-density carrier gas (c) interacting with a phonon bath (p), the explicit form of the global Hamiltonian in Equation (5) is given by

(15) $$\hat{H}={\hat{H}}_{{}^{\circ}}+{\hat{H}}^{\prime }=\left({\hat{H}}_{c}+{\hat{H}}_{p}\right)\;+\;\left({\hat{H}}_{\mathrm{cp}}\right)\;,$$

where

(16) $${\hat{H}}_{c}=\sum_{\alpha }|\alpha \rangle \epsilon_{\alpha }\langle \alpha |$$

is the one-electron term,

(17) $${\hat{H}}_{p}=\sum_{q}\epsilon_{q}{\hat{b}}_{q}^{†}{\hat{b}}_{q}$$

describes the system of noninteracting phonons with wavevector q and energy $\epsilon_{q}$, and

(18) $${\hat{H}}_{\mathrm{cp}}=\sum_{\alpha \alpha^{\prime }}|\alpha \rangle \left[\sum_{q}\left(g_{\alpha \alpha^{\prime }}^{q,-}{\hat{b}}_{q}+g_{\alpha^{\prime }\alpha }^{q,+}{\hat{b}}_{q}^{†}\right)\right]\langle \alpha^{\prime }|$$

is the (one-electron) carrier–phonon interaction term. Here, $\epsilon_{\alpha }$ is the energy level corresponding to the generic one-electron eigenstate |α〉; − and + refer, respectively, to phonon absorption and emission; while the explicit form of the coupling matrix elements $g_{\alpha \alpha^{\prime }}^{q,\pm }=g_{\alpha \alpha^{\prime }}^{q,\mp *}$ depends on the particular phonon branch (acoustic, optical, etc.) as well as on the coupling mechanism considered (deformation potential, polar coupling, etc.).

Adopting the one-electron picture in Equation (14) and employing the explicit form of the global (carrier plus phonon) Hamiltonian in Equation (15), it is possible to derive the following scattering superoperator acting on the one-electron density matrix $\rho_{\alpha_{1}\alpha_{2}}$ [7]:

(19) $${\left.\frac{d\rho_{\alpha_{1}\alpha_{2}}}{dt}\right|}_{\mathrm{inco}}=\frac{1}{2}\sum_{\alpha_{1}^{\prime }\alpha_{2}^{\prime }}\left(P_{\alpha_{1}\alpha_{2},\alpha_{1}^{\prime }\alpha_{2}^{\prime }}^{cp}\rho_{\alpha_{1}^{\prime }\alpha_{2}^{\prime }}-P_{\alpha_{1}^{\prime }\alpha_{1}^{\prime },\alpha_{1}\alpha_{2}^{\prime }}^{cp\;*}\rho_{\alpha_{2}^{\prime }\alpha_{2}}\right)+H.c.$$

with generalized carrier–phonon scattering rates

(20) $$P_{\alpha_{1}\alpha_{2},\alpha_{1}^{\prime }\alpha_{2}^{\prime }}^{cp}=\sum_{q,\pm }a_{\alpha_{1}\alpha_{1}^{\prime }}^{q,\pm }b_{\alpha_{2}\alpha_{2}^{\prime }}^{q,\pm *}\;,$$

where

(21) $$a_{\alpha \alpha^{\prime }}^{q,\pm }=\sqrt{\frac{2\pi \left(n_{q}^{{}^{\circ}}+\frac{1}{2}\pm \frac{1}{2}\right)}{\hbar }}g_{\alpha \alpha^{\prime }}^{q,\pm }\;,\;b_{\alpha \alpha^{\prime }}^{q,\pm }=a_{\alpha \alpha^{\prime }}^{q,\pm }\delta \left(\epsilon_{\alpha }-\epsilon_{\alpha^{\prime }}\pm \epsilon_{q}\right)\;,$$

and $n_{q}^{{}^{\circ}}$ denotes the equilibrium phonon distribution.

It is worth stressing that the diagonal elements ($\alpha_{1}\alpha_{1}^{\prime }=\alpha_{2}\alpha_{2}^{\prime }$) of the generalized rates in Equation (20) coincide once again with the usual Fermi’s-golden-rule prescription applied to carrier–phonon interaction [7]:

(22) $$P_{\alpha \to \alpha^{\prime }}\equiv P_{\alpha^{\prime }\alpha^{\prime },\alpha \alpha }^{cp}=\frac{2\pi }{\hbar }\sum_{q,\pm }{\left|g_{\alpha^{\prime }\alpha }^{q,\pm }\right|}^{2}\delta \left(\epsilon_{\alpha^{\prime }}-\epsilon_{\alpha }\pm \epsilon_{q}\right)\;.$$

To investigate the impact of possible positivity violations induced by the conventional (i.e., non-Lindblad) adiabatic treatment reviewed so far, we consider a prototypical quantum-dot nanostructure described again via the simple two-level system introduced in Section 1. The latter is characterized by two electronic states only (α≡{a,b}) with an energy splitting $\Delta_{c}=\epsilon_{b}-\epsilon_{a}$ and is described by the two-by-two density matrix in Equation (1). Moreover, regardless of the specific phonon mode considered, we shall adopt q-independent coupling matrices of the following form:

(23) $$\left(\begin{array}{cc} g_{bb}^{q,\pm } & g_{ba}^{q,\pm }\\ g_{ab}^{q,\pm } & g_{aa}^{q,\pm } \end{array}\right)=\left(\begin{array}{cc} 0 & g\\ g & 0 \end{array}\right)\;.$$

This corresponds to neglecting diagonal coupling terms (a→a and b→b); the related phonon-induced energy renormalizations are relatively small in conventional solid-state systems and have negligible impact on energy dissipation and decoherence phenomena.

As anticipated in Section 1, in this particular case, the basic positivity requirement amounts to asking that whether the eigenvalues $\Lambda_{\pm }$ in Equation (4) or, equivalently, the determinant $f_{a}f_{b}-{\left|p\right|}^{2}$ are nonnegative. In order to check the positivity of the density matrix, its eigenvalue analysis is therefore imperative; more specifically, since $\Lambda_{+}\geq \Lambda_{-}$, it is enough to check the nonnegativity of the eigenvalue $\Lambda_{-}$.

For the simulated experiments presented here below, we are assuming as an initial condition a low-density Bell state, namely $f_{a}\left(0\right)=f_{b}\left(0\right)=p\left(0\right)\ll 1$, and investigating energy dissipation and decoherence induced on the two-level system by an acoustic-like phonon mode, i.e., a linear-dispersion mode characterized by a bandwidth $0\leq \epsilon_{q}\leq \Delta_{p}$ much greater than the interlevel splitting $\Delta_{c}$.

**Figure 2:**
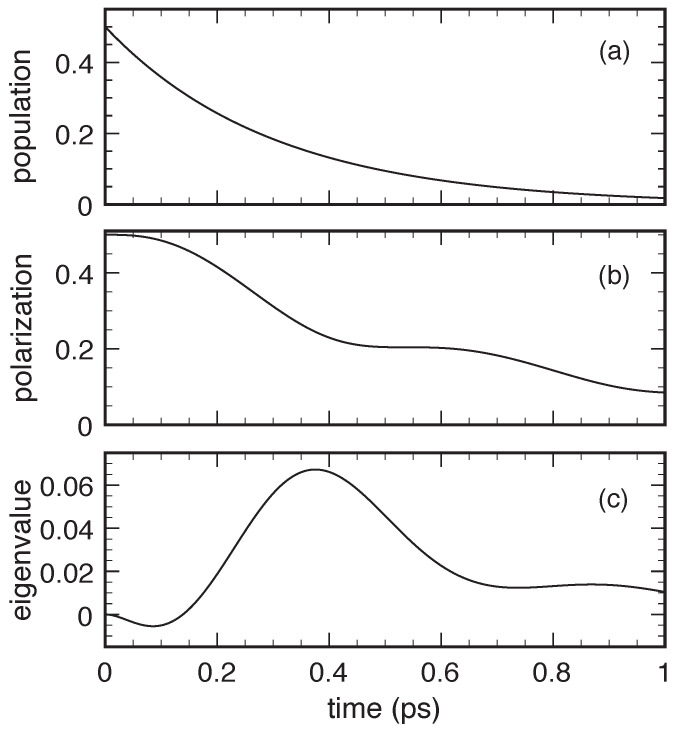
Energy dissipation and decoherence for an electronic two-level system ($\Delta_{c}=4$ meV) coupled to an acoustic-like phonon mode ($\Delta_{\mathrm{cp}}≃2$ meV) in the low-temperature and low-density limit: Excited-level relative population $f_{b}/\left(f_{a}+f_{b}\right)$ (upper panel), relative interlevel-polarization modulus $\left|p|/(f_{a}+f_{b}\right)$ (middle panel), and relative eigenvalue $\Lambda_{-}/\left(f_{a}+f_{b}\right)$ (lower panel) as a function of time obtained via the Markovian dissipation model in Equation (19) equipped with the non-Lindblad rates in Equation (20).

Figure 2 shows the results obtained via the Markovian dissipation model in Equation (19) equipped with the non-Lindblad scattering rates in Equation (20) in the low-density and low-temperature limit for $\Delta_{c}=4$ meV and for a relatively strong carrier–phonon interaction. More specifically, in order to mimic carrier-acoustic phonon scattering in GaN-based nanomaterials, the coupling coefficient *g* in Equation (23) and the phonon velocity have been chosen such to produce a semiclassical scattering time $P_{b\to a}^{-1}=0.3$ ps (see Equation (22)), which corresponds to an effective interlevel coupling energy $\Delta_{\mathrm{cp}}\equiv \hbar P_{b\to a}$ of about 2 meV. For the above system parameters, we deal with a dimensionless coupling coefficient $\eta =\Delta_{\mathrm{cp}}/\Delta_{c}=0.5$. Such a relatively strong carrier–phonon coupling is indeed typical of GaN-based quantum-dot structures. As we can see, in spite of the typical energy relaxation versus decoherence scenario reported in the upper (population) and middle (polarization) panels, here, the lower panel unequivocally displays negative values (though extremely small) of the density-matrix eigenvalue $\Lambda_{-}$.

**Figure 3:**
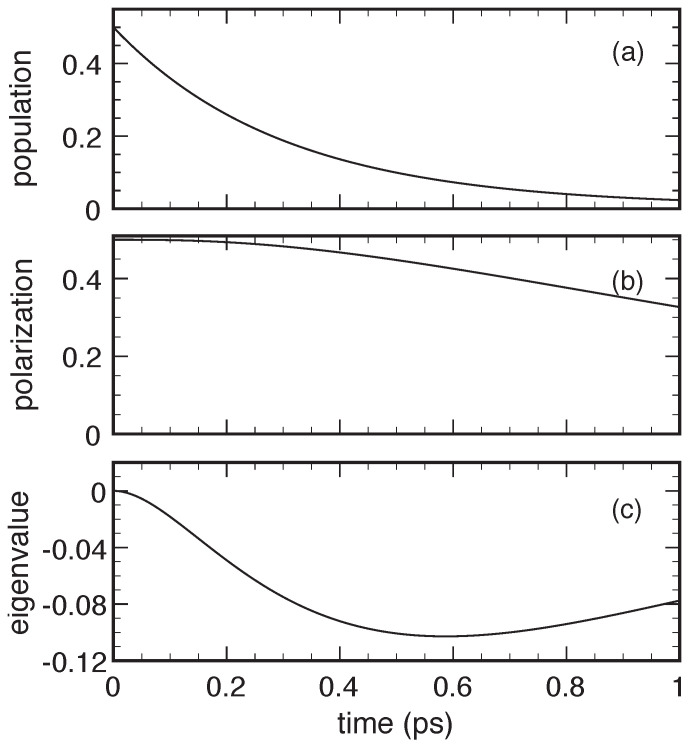
Same as in Figure 2 but for a smaller interlevel energy splitting ($\Delta_{c}=1$ meV).

To show that the small/negligible positivity violations reported in Figure 2 may also become much larger and thus highly problematic, we have repeated the simulated experiment in Figure 2, reducing the interlevel energy splitting by a factor of four, $\Delta_{c}=1$ meV, which amounts to increasing the coupling coefficient η up to 2. The results are reported in Figure 3. As one can see, in spite of a very similar population dynamics (compare upper panels in Figure 2 and Figure 3), we deal with a significant slowdown of the polarization decay (middle panels). Moreover the density-matrix eigenvalue $\Lambda_{-}$ is negative (lower panel) and is an absolute value, now nearly comparable with the typical population and polarization ones. The new simulated experiment in Figure 3 constitutes a non-ambiguous proof that positivity violations, typical of non-Lindblad Markov models, may constitute a severe limitation in the study of solid-state quantum materials and related devices characterized by a strong carrier–phonon coupling, like, e.g., GaN-based quantum-dot nanostructures.

A closer inspection shows that the physical origin of the above positivity violations is an anomalous underestimation of decoherence processes, leading to a slowdown of the polarization decay. This, in turn, leads to negative values of the density-matrix determinant ($f_{a}f_{b}-{\left|p\right|}^{2}$) and thus to negative eigenvalues. This limitation is indeed a peculiar feature of the non-Lindblad Markov treatments reviewed so far, and can be easily avoided adopting the alternative (Lindblad-type) adiabatic-decoupling scheme presented below.

### 2.2. Lindblad-Type Adiabatic-Decoupling Scheme

As anticipated, the choice of the adiabatic-decoupling scheme is definitely not unique. Compared to the conventional approach recalled so far, the alternative adiabatic scheme proposed in Reference [19], based on a time symmetrization between microscopic and macroscopic scales, enables one to replace the non-Lindblad incoherent contribution in Equation (6) with the following Lindblad superoperator [7]:

(24) $${\left.\frac{d\hat{\rho }}{dt}\right|}_{\mathrm{inco}}=\sum_{s}\left({\hat{A}}^{s}\hat{\rho }{\hat{A}}^{s†}-\frac{1}{2}\left\{{\hat{A}}^{s†}{\hat{A}}^{s},\hat{\rho }\right\}\right)$$

with

(25) $${\hat{A}}^{s}=\lim_{\bar{\epsilon }\to 0}{\left(\frac{2{\bar{\epsilon }}^{2}}{\pi \hbar^{6}}\right)}^{\frac{1}{4}}\int_{-\infty }^{+\infty }e^{-\frac{{\hat{H}}_{{}^{\circ}}t^{\prime }}{i\hbar }}{\hat{H}}_{s}^{\prime }e^{\frac{{\hat{H}}_{{}^{\circ}}t^{\prime }}{i\hbar }}e^{-{\left(\frac{\bar{\epsilon }t^{\prime }}{\hbar }\right)}^{2}}dt^{\prime }\;.$$

As discussed in Reference [19], here, $\bar{\epsilon }$ plays the role of an energy broadening induced by a finite collision duration and/or to a finite single-particle life-time [19].

The new dissipation model in Equation (24) can still be expressed via the global density-matrix superoperator in Equation (8) provided to replace the non-Lindblad rates in Equation (9) with the following (Lindblad-type) version:

(26) $$P_{i_{1}i_{2},i_{1}^{\prime }i_{2}^{\prime }}^{s}=A_{i_{1}i_{1}^{\prime }}^{s}A_{i_{2}i_{2}^{\prime }}^{s*}\;.$$

While their diagonal (i.e., semiclassical) elements ($i_{1}i_{1}^{\prime }=i_{2}i_{2}^{\prime }$) coincide again with the standard Fermi’s-golden-rule prescription in Equation (10), the new (Lindblad) version in Equation (26) exhibits a more symmetric structure, a clear fingerprint of the time symmetrization previously mentioned.

Starting from the Lindblad-type global superoperator in Equation (24) and adopting once again the carrier–phonon mean-field approximation via the factorization scheme in Equation (12), it is possible to get an effective dissipation model for the reduced density-matrix operator ${\hat{\rho }}_{c}$, which is still of Lindblad type [7]:

(27) $${\left.\frac{d{\hat{\rho }}_{c}}{dt}\right|}_{\mathrm{inco}}=\sum_{s}\left({\hat{A}}_{c}^{s}{\hat{\rho }}_{c}{\hat{A}}_{c}^{s†}-\frac{1}{2}\left\{{\hat{A}}_{c}^{s†}{\hat{A}}_{c}^{s},{\hat{\rho }}_{c}\right\}\right)\;.$$

The explicit form of the new electronic operators ${\hat{A}}_{c}^{s}$ can be derived starting from the global Lindblad operators ${\hat{A}}^{s}$ in Equation (25). Once again, in spite of their very same formal structure, Equations (24) and (27) describe the system dynamics at different levels.

Adopting once again the one-electron picture previously introduced (see Equation (14)) and applying the Lindblad treatment recalled so far to the case of the carrier-plus-phonon system in Equation (15), it is possible to get the very same density-matrix superoperator in Equation (19) provided to replace the non-Lindblad carrier–phonon rates in Equation (20) with the following Lindblad-type version [7]:

(28) $$P_{\alpha_{1}\alpha_{2},\alpha_{1}^{\prime }\alpha_{2}^{\prime }}=\sum_{q,\pm }A_{\alpha_{1}\alpha_{1}^{\prime }}^{q,\pm }A_{\alpha_{2}\alpha_{2}^{\prime }}^{q,\pm *}\;,$$

where

(29) $$A_{\alpha \alpha^{\prime }}^{q,\pm }=\sqrt{\frac{2\pi \left(n_{q}^{{}^{\circ}}+\frac{1}{2}\pm \frac{1}{2}\right)}{\hbar }}g_{\alpha \alpha^{\prime }}^{q,\pm }D_{\alpha \alpha^{\prime }}^{q,\pm }$$

and

(30) $$D_{\alpha \alpha^{\prime }}^{q,\pm }=\lim_{\bar{\epsilon }\to 0}\frac{e^{-{\left(\frac{\epsilon_{\alpha }-\epsilon_{\alpha^{\prime }}\pm \epsilon_{q}}{2\bar{\epsilon }}\right)}^{2}}}{{\left(2\pi {\bar{\epsilon }}^{2}\right)}^{\frac{1}{4}}}\;.$$

We stress that the diagonal elements ($\alpha_{1}\alpha_{1}^{\prime }=\alpha_{2}\alpha_{2}^{\prime }$) of the new (Lindblad-type) carrier–phonon rates in Equation (28) coincide once again with the usual Fermi’s-golden-rule prescription in Equation (22).

**Figure 4:**
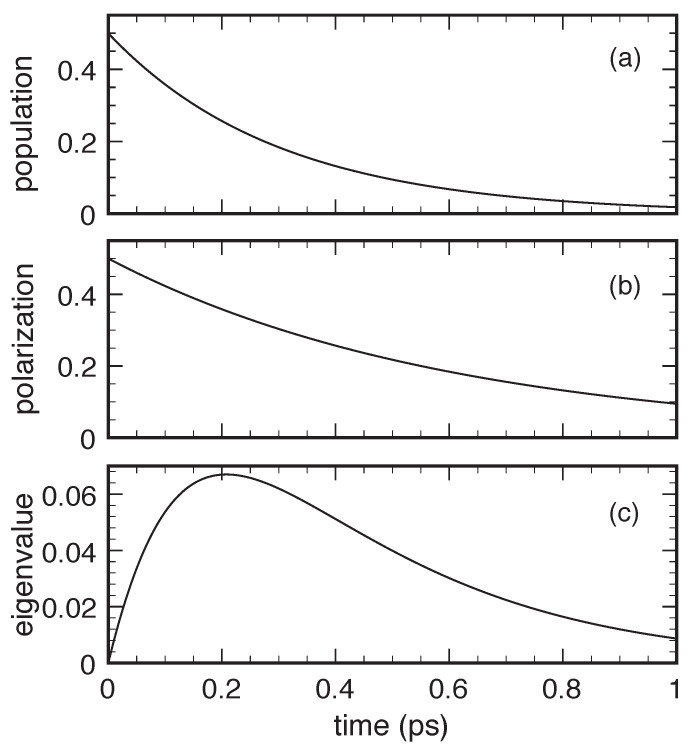
Same as in Figure 2 but replacing the non-Lindblad carrier–phonon scattering rates in Equation (20) with the Lindblad ones in Equation (28).

**Figure 5:**
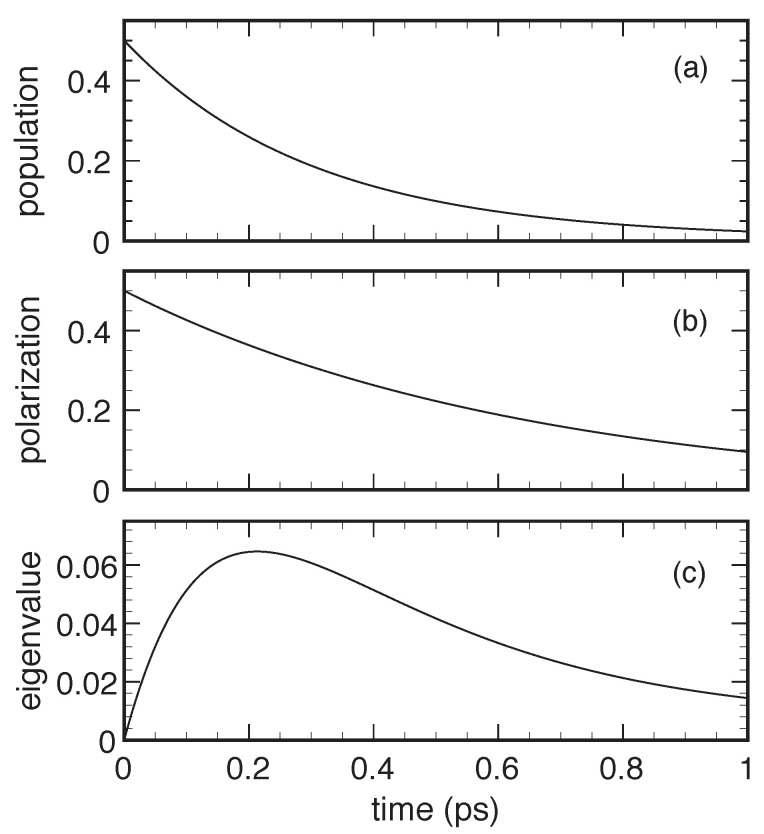
Same as in Figure 3 but replacing the non-Lindblad carrier–phonon scattering rates in Equation (20) with the Lindblad ones in Equation (28).

To concretely test the quality of the alternative adiabatic-decoupling scheme reviewed so far, we have repeated the previous simulated experiments in Figure 2 and Figure 3, replacing the non-Lindblad carrier–phonon scattering rates in Equation (20) with the Lindblad ones in Equation (28). The new results, reported in Figure 4 and Figure 5, fully confirm the absence of positivity violations (see the nonnegative eigenvalue profiles in the lower panels), as expected for any Lindblad-type scattering model. A closer comparison between the former (non-Lindblad) and the present (Lindblad) simulations shows that, in spite of a very similar population dynamics, the Lindblad-type dissipation model is always characterized by a faster polarization decay, thus preventing in any case the positivity violations of the non-Lindblad one reported in Figure 2 and Figure 3.

### 2.3. Generalization to the Nonlinear Regime

As anticipated, while in the low-density limit, dissipation versus decoherence phenomena can safely be described via the simple one-electron picture previously introduced, at high carrier concentrations, a genuine many-body treatment is imperative [7]; this is typically accomplished via the so-called single-particle picture, based on a mean-field (or Hartree–Fock) treatment of our many-electron system. Indeed, many of the physical quantities of interest in the study of solid-state quantum devices are described via single-particle electronic operators of the following form:

(31) $${\hat{G}}_{c}=\sum_{\alpha_{1}\alpha_{2}}G_{\alpha_{1}\alpha_{2}}{\hat{c}}_{\alpha_{1}}^{†}{\hat{c}}_{\alpha_{2}}\;,$$

where ${\hat{c}}_{\alpha }^{†}$ and ${\hat{c}}_{\alpha }$ are the usual creation and destruction operators over the electronic single-particle states |α〉. Recalling that, for any electronic operator, one has $\langle G_{c}\rangle =\mathrm{tr}\left\{\hat{\rho }{\hat{G}}_{c}\right\}=\mathrm{tr}{\left\{{\hat{\rho }}_{c}{\hat{G}}_{c}\right\}}_{c}$, the average value of the single-particle operator in Equation (31) can be written as

(32) $$\langle G_{c}\rangle =\sum_{\alpha_{1}\alpha_{2}}\rho_{\alpha_{1}\alpha_{2}}G_{\alpha_{2}\alpha_{1}}$$

where

(33) $$\rho_{\alpha_{1}\alpha_{2}}=\mathrm{tr}{\left\{{\hat{c}}_{\alpha_{2}}^{†}{\hat{c}}_{\alpha_{1}}{\hat{\rho }}_{c}\right\}}_{c}$$

is the single-particle density matrix. It is worth stressing that, in the low-density limit, the single- particle density matrix in Equation (33) is equivalent to the one-electron density matrix introduced in Equation (14).

To study the time evolution of single-particle quantities, such as total carrier density, mean kinetic energy, charge current, and so on, it is then vital to derive a closed equation of motion for the above single-particle density matrix. Combining its definition in Equation (33) with the many-electron Lindblad dynamics in Equation (27) and employing the cyclic property of the trace, one gets

(34) $${\left.\frac{d\rho_{\alpha_{1}\alpha_{2}}}{dt}\right|}_{\mathrm{inco}}=\frac{1}{2}\sum_{s}\mathrm{tr}{\left\{\left[{\hat{A}}_{c}^{s†},{\hat{c}}_{\alpha_{2}}^{†}{\hat{c}}_{\alpha_{1}}\right]{\hat{A}}_{c}^{s}{\hat{\rho }}_{c}\right\}}_{c}\;+\;H.c.\;$$

To derive a closed equation of motion for the single-particle density matrix, it is now crucial to specify the form/structure of the many-electron Lindblad operators ${\hat{A}}_{c}^{s}$ in Equation (27), which, in turn, is dictated by the specific interaction mechanism considered.

For the case of a generic carrier–phonon interaction mechanism, the corresponding (one-body) Lindblad operator is always of the following form:

(35) $${\hat{A}}_{c}^{s}=\sum_{\alpha \alpha^{\prime }}A_{\alpha \alpha^{\prime }}^{\mathrm{cp}}{\hat{c}}_{\alpha }^{†}{\hat{c}}_{\alpha^{\prime }}\;.$$

Equation (35) describes the phonon-induced carrier transition from the initial state $\alpha^{\prime }$ to the final state α. In this case, the label s=q,± corresponds to the emission (+) or absorption (−) of a phonon with wavevector q. By inserting the carrier–phonon Lindblad operator in Equation (35) into Equation (34) and by employing the fermionic anticommutation relations, it is easy to show [21] that the contribution to the system dynamics due to the generic carrier–phonon interaction mechanism (s=cp) involves average values of four fermionic operators of the following form:

(36) $$h_{\alpha_{3}\alpha_{4},\alpha_{3}^{\prime }\alpha_{4}^{\prime }}=\mathrm{tr}{\left\{{\hat{c}}_{\alpha_{3}}^{†}{\hat{c}}_{\alpha_{4}}{\hat{c}}_{\alpha_{3}^{\prime }}^{†}{\hat{c}}_{\alpha_{4}^{\prime }}{\hat{\rho }}_{c}\right\}}_{c}\;.$$

For the carrier–carrier interaction (s=cc), the Lindblad operator has the general two-body structure:

(37) $${\hat{A}}_{c}^{s}=\frac{1}{2}\sum_{\alpha \bar{\alpha },\alpha^{\prime }{\bar{\alpha }}^{\prime }}A_{\alpha \bar{\alpha },\alpha^{\prime }{\bar{\alpha }}^{\prime }}^{\mathrm{cc}}{\hat{c}}_{\alpha }^{†}{\hat{c}}_{\bar{\alpha }}^{†}{\hat{c}}_{{\bar{\alpha }}^{\prime }}{\hat{c}}_{\alpha^{\prime }}\;,$$

The latter describes the transition of the electronic pair from the initial (two-body) state $\alpha^{\prime }{\bar{\alpha }}^{\prime }$ to the final state $\alpha \bar{\alpha }$. As shown in Reference [21], by inserting Equation (37) into Equation (34), the contribution to the system dynamics due to carrier–carrier interaction (s=cc) involves average values of eight fermionic operators of the following form:

(38) $$k_{\alpha_{5}\alpha_{6}\alpha_{7}\alpha_{8},\alpha_{5}^{\prime }\alpha_{6}^{\prime }\alpha_{7}^{\prime }\alpha_{8}^{\prime }}\;=\;\mathrm{tr}{\left\{{\hat{c}}_{\alpha_{5}}^{†}{\hat{c}}_{\alpha_{6}}^{†}{\hat{c}}_{\alpha_{7}}{\hat{c}}_{\alpha_{8}}{\hat{c}}_{\alpha_{5}^{\prime }}^{†}{\hat{c}}_{\alpha_{6}^{\prime }}^{†}{\hat{c}}_{\alpha_{7}^{\prime }}{\hat{c}}_{\alpha_{8}^{\prime }}{\hat{\rho }}_{c}\right\}}_{c}\;.$$

As anticipated, the key step in getting a closed equation of motion for the single-particle density matrix is to adopt the well-known mean-field (or correlation-expansion) approximation [65]; as discussed in Reference [21], employing this approximation scheme and omitting renormalization terms [65], for both carrier–phonon and carrier–carrier scattering, the resulting single-particle equation is given by

(39) $${\left.\frac{d\rho_{\alpha_{1}\alpha_{2}}}{dt}\right|}_{\mathrm{inco}}=\frac{1}{2}\sum_{\alpha^{\prime }\alpha_{1}^{\prime }\alpha_{2}^{\prime }}\left(\left(\delta_{\alpha_{1}\alpha^{\prime }}-\rho_{\alpha_{1}\alpha^{\prime }}\right)P_{\alpha^{\prime }\alpha_{2},\alpha_{1}^{\prime }\alpha_{2}^{\prime }}^{s}\rho_{\alpha_{1}^{\prime }\alpha_{2}^{\prime }}-\left(\delta_{\alpha^{\prime }\alpha_{1}^{\prime }}-\rho_{\alpha^{\prime }\alpha_{1}^{\prime }}\right)P_{\alpha^{\prime }\alpha_{1}^{\prime },\alpha_{1}\alpha_{2}^{\prime }}^{s*}\rho_{\alpha_{2}^{\prime }\alpha_{2}}\right)\;+\;H.c.$$

with generalized carrier–phonon scattering rates

(40) $$P_{\alpha_{1}\alpha_{2},\alpha_{1}^{\prime }\alpha_{2}^{\prime }}^{s=\mathrm{cp}}=A_{\alpha_{1}\alpha_{1}^{\prime }}^{\mathrm{cp}}A_{\alpha_{2}\alpha_{2}^{\prime }}^{\mathrm{cp}*}$$

and generalized carrier–carrier scattering rates

(41) $$P_{\alpha_{1}\alpha_{2},\alpha_{1}^{\prime }\alpha_{2}^{\prime }}^{s=\mathrm{cc}}=2\sum_{{\bar{\alpha }}_{1}{\bar{\alpha }}_{2},{\bar{\alpha }}_{1}^{\prime }{\bar{\alpha }}_{2}^{\prime }}\left(\delta_{{\bar{\alpha }}_{2}{\bar{\alpha }}_{1}}-\rho_{{\bar{\alpha }}_{2}{\bar{\alpha }}_{1}}\right)A_{\alpha_{1}{\bar{\alpha }}_{1},\alpha_{1}^{\prime }{\bar{\alpha }}_{1}^{\prime }}^{\mathrm{cc}}A_{\alpha_{2}{\bar{\alpha }}_{2},\alpha_{2}^{\prime }{\bar{\alpha }}_{2}^{\prime }}^{\mathrm{cc}*}\rho_{{\bar{\alpha }}_{1}^{\prime }{\bar{\alpha }}_{2}^{\prime }}\;,$$

where

(42) $$A_{\alpha \bar{\alpha },\alpha^{\prime }{\bar{\alpha }}^{\prime }}^{\mathrm{cc}}\;=\;\frac{1}{4}\left(A_{\alpha \bar{\alpha },\alpha^{\prime }{\bar{\alpha }}^{\prime }}^{\mathrm{cc}}\;-\;A_{\bar{\alpha }\alpha ,\alpha^{\prime }{\bar{\alpha }}^{\prime }}^{\mathrm{cc}}\;-\;A_{\alpha \bar{\alpha },{\bar{\alpha }}^{\prime }\alpha^{\prime }}^{\mathrm{cc}}\;+\;A_{\bar{\alpha }\alpha ,{\bar{\alpha }}^{\prime }\alpha^{\prime }}^{\mathrm{cc}}\right)$$

denote the totally antisymmetric parts of the two-body coefficients in Equation (37).

We stress that, opposite to the carrier–phonon rates in Equation (40), the generalized carrier–carrier rates in (41) are themselves a function of the single-particle density matrix; this is a clear fingerprint of the two-body nature of the carrier–carrier interaction (see below).

The single-particle scattering superoperator in Equation (39) is the result of positive-like (in-scattering) and negative-like (out-scattering) contributions, which are nonlinear functions of the single-particle density matrix. Indeed, in the semiclassical limit [7], namely $\rho_{\alpha_{1}\alpha_{2}}=f_{\alpha_{1}}\delta_{\alpha_{1}\alpha_{2}}$, the density-matrix equation Equation (39) assumes the expected nonlinear Boltzmann-type form:

(43) $${\left.\frac{df_{\alpha }}{dt}\right|}_{\mathrm{inco}}=\sum_{\alpha^{\prime }}\left(\left(1-f_{\alpha }\right)P_{\alpha \alpha^{\prime }}^{s}f_{\alpha^{\prime }}-\left(1-f_{\alpha^{\prime }}\right)P_{\alpha^{\prime }\alpha }^{s}f_{\alpha }\right)$$

with semiclassical carrier–phonon scattering rates

(44) $$P_{\alpha \alpha^{\prime }}^{s=\mathrm{cp}}=P_{\alpha \alpha ,\alpha^{\prime }\alpha^{\prime }}^{s=\mathrm{cp}}={\left|A_{\alpha \alpha^{\prime }}^{\mathrm{cp}}\right|}^{2}$$

and semiclassical carrier–carrier scattering rates

(45) $$P_{\alpha \alpha^{\prime }}^{s=\mathrm{cc}}=P_{\alpha \alpha ,\alpha^{\prime }\alpha^{\prime }}^{s=\mathrm{cc}}=2\sum_{\bar{\alpha }{\bar{\alpha }}^{\prime }}\left(1-f_{\bar{\alpha }}\right){\left|A_{\alpha \bar{\alpha },\alpha^{\prime }{\bar{\alpha }}^{\prime }}^{\mathrm{cc}}\right|}^{2}f_{{\bar{\alpha }}^{\prime }}\;.$$

The above semiclassical limit clearly shows that the nonlinearity factors $\left(\delta_{\alpha_{1}\alpha_{2}}-\rho_{\alpha_{1}\alpha_{2}}\right)$ in Equation (39) as well as in Equation (41) can be regarded as the quantum-mechanical generalization of the Pauli factors $\left(1-f_{\alpha }\right)$ of the conventional Boltzmann theory.

A closer inspection of Equations (39) and (41) as well as of their semiclassical counterparts in Equations (43) and (45) confirms the two-body nature of the carrier–carrier interaction. Indeed, differently from the carrier–phonon scattering, in this case, the density-matrix equation describes the time evolution of a so-called “main carrier” α interacting with a so-called “partner carrier” $\bar{\alpha }$.

Let us finally face the key issue related to the present single-particle treatment, namely the positivity analysis of the nonlinear density-matrix equation in Equation (39). Indeed, for a physical state, the eigenvalues of the single-particle density matrix $\rho_{\alpha_{1}\alpha_{2}}$ are necessarily nonnegative but also smaller than one (i.e., Pauli exclusion principle); to preserve such physical nature, it is vital to verify that the scattering-induced time evolution maintains the values of the eigenvalues within the interval [0, 1].

To this end, let us start from the case of carrier–phonon interaction previously discussed, for which the nonlinear equation in Equation (39) (equipped with the generalized rates in Equation (40)) can also be written in a more compact way via the one-electron operators:

(46) $$\hat{\rho }=\sum_{\alpha_{1}\alpha_{2}}|\alpha_{1}\rangle \rho_{\alpha_{1}\alpha_{2}}\langle \alpha_{2}|$$

and

(47) $$\hat{A}=\sum_{\alpha_{1}\alpha_{2}}|\alpha_{1}\rangle A_{\alpha_{1}\alpha_{2}}^{\mathrm{cp}}\langle \alpha_{2}|$$

as

(48) $${\left.\frac{d\hat{\rho }}{dt}\right|}_{\mathrm{inco}}=\frac{1}{2}\left(\left(\hat{I}-\hat{\rho }\right)\hat{A}\hat{\rho }{\hat{A}}^{†}-{\hat{A}}^{†}\left(\hat{I}-\hat{\rho }\right)\hat{A}\hat{\rho }\right)\;+\;H.c.\;,$$

where $\hat{I}$ denotes the identity operator of the one-electron Hilbert space. We stress that, due to the quantum-mechanical Pauli factors $\left(\hat{I}-\hat{\rho }\right)$, the above scattering superoperator in Equation (48) is nonlinear in $\hat{\rho }$ and thus is definitely non-Lindblad. Only in the low-density limit, i.e., $\hat{I}-\hat{\rho }\to \hat{I}$, the nonlinear equation in Equation (48) reduces to the Lindblad superoperator

(49) $${\left.\frac{d\hat{\rho }}{dt}\right|}_{\mathrm{inco}}=\hat{A}\hat{\rho }{\hat{A}}^{†}-\frac{1}{2}\left\{{\hat{A}}^{†}\hat{A},\hat{\rho }\right\}\;,$$

and the positive-definite character of $\hat{\rho }$ is thereby ensured.

For high-density conditions, in contrast, no straightforward conclusion can be drawn about the positive-definite character of the corresponding time evolution. Nevertheless, it is easy to show [21] that the nonlinear single-particle equation in Equation (39) does preserve the positive-definite character of $\hat{\rho }$. To this end, let us consider the instantaneous (i.e., time-dependent) eigenvalues $\Lambda_{\lambda }$ and eigenvectors |λ〉 of the density-matrix operator, i.e.,

(50) $$\hat{\rho }|\lambda \rangle =\Lambda_{\lambda }|\lambda \rangle \;,$$

which implies that

(51) $$\Lambda_{\lambda }=\langle \lambda |\hat{\rho }|\lambda \rangle \;.$$

As anticipated, the eigenvalues in Equation (50) corresponding to a physical state are necessarily nonnegative as well as smaller than one. The scattering-induced time evolution should therefore maintain the values of the $\Lambda_{\lambda }$ within the physical interval [0, 1]; this can be verified by studying the time derivative of the generic eigenvalue in Equation (51):

(52) $$\frac{d\Lambda_{\lambda }}{dt}=\frac{d\langle \lambda |}{dt}\hat{\rho }|\lambda \rangle +\langle \lambda |\frac{d\hat{\rho }}{dt}|\lambda \rangle +\langle \lambda |\hat{\rho }\frac{d|\lambda \rangle }{dt}\;.$$

Thanks to the completeness of the basis set {|λ〉}, the time derivative in Equation (52) can also be written as follows:

$$\frac{d\Lambda_{\lambda }}{dt}=\sum_{\lambda^{\prime }}\frac{d\langle \lambda |}{dt}|\lambda^{\prime }\rangle \langle \lambda^{\prime }|\hat{\rho }|\lambda \rangle +\langle \lambda |\frac{d\hat{\rho }}{dt}|\lambda \rangle +\sum_{\lambda^{\prime }}\langle \lambda |\hat{\rho }|\lambda^{\prime }\rangle \langle \lambda^{\prime }|\frac{d|\lambda \rangle }{dt}\;.$$

Recalling that

(53) $$\langle \lambda |\hat{\rho }|\lambda^{\prime }\rangle =\Lambda_{\lambda }\delta_{\lambda \lambda^{\prime }}\;,$$

the result in Equation (53) reduces to

(54) $$\frac{d\Lambda_{\lambda }}{dt}=\Lambda_{\lambda }\frac{d\langle \lambda |}{dt}|\lambda \rangle +\langle \lambda |\frac{d\hat{\rho }}{dt}|\lambda \rangle +\Lambda_{\lambda }\langle \lambda |\frac{d|\lambda \rangle }{dt}\;.$$

Taking into account that $\frac{d\langle \lambda |\lambda \rangle }{dt}=0\;,$ the first and third terms on the right hand side of Equation (54) cancel out exactly, and one finally gets

(55) $$\frac{d\Lambda_{\lambda }}{dt}=\frac{d\rho_{\lambda \lambda }}{dt}\;.$$

This tells us that the time variation of the eigenvalues $\Lambda_{\lambda }$ coincides with the time variation of the diagonal elements $\rho_{\lambda \lambda }$ of the operator $\hat{\rho }$ within the instantaneous eigenbasis {|λ〉}.

To study the above time derivative, the key step is to examine the explicit form of the single-particle scattering superoperator written in the density-matrix eigenbasis in Equation (50). Taking into account that the generic density-matrix equation in Equation (39) is basis-independent, by replacing the original single-particle basis {|α〉} with the density-matrix eigenbasis {|λ〉} and making use of Equation (53), its diagonal elements turn out to be as follows:

(56) $$\frac{d\rho_{\lambda \lambda }}{dt}=\sum_{\lambda^{\prime }}\left[\left(1-\Lambda_{\lambda }\right)P_{\lambda \lambda^{\prime }}^{s}\Lambda_{\lambda^{\prime }}-\left(1-\Lambda_{\lambda^{\prime }}\right)P_{\lambda^{\prime }\lambda }^{s}\Lambda_{\lambda }\right]\;,$$

where

(57) $$P_{\lambda \lambda^{\prime }}^{s}=P_{\lambda \lambda ,\lambda^{\prime }\lambda^{\prime }}^{s}$$

are positive-definite quantities given by the diagonal elements of the generalized scattering rates (see Equations (40) and (41)) written in our instantaneous density-matrix eigenbasis. By inserting this last result into Equation (55), we finally get

(58) $$\frac{d\Lambda_{\lambda }}{dt}=\sum_{\lambda^{\prime }}\left[\left(1-\Lambda_{\lambda }\right)P_{\lambda \lambda^{\prime }}^{s}\Lambda_{\lambda^{\prime }}-\left(1-\Lambda_{\lambda^{\prime }}\right)P_{\lambda^{\prime }\lambda }^{s}\Lambda_{\lambda }\right]\;.$$

This result is highly nontrivial: it states that, in spite of the partially coherent nature of the carrier dynamics in Equation (39), the time evolution of the eigenvalues $\Lambda_{\lambda }$ is governed by a nonlinear Boltzmann-type equation, formally identical to the semiclassical result in Equation (43).

We are then finally able to state that the physical interval [0, 1] is the only possible variation range for the eigenvalues $\Lambda_{\lambda }$. Indeed, it is easy to show [21] that, when the latter approach the extremal values, 0 or 1, their time derivatives do not allow them to exit the interval. In particular, a closer inspection of the Boltzmann-like equation in Equation (58) shows the following:

(i) if one of the eigenvalues $\Lambda_{\lambda }$ is equal to zero, the corresponding time derivative in Equation (58) is always nonnegative;
(ii) if one of the eigenvalues $\Lambda_{\lambda }$ is equal to one, its time derivative in Equation (58) is always nonpositive.

This leads us to the important conclusion that, for both carrier–phonon and carrier–carrier scattering, the nonlinear single-particle equation in Equation (39) preserves the positive-definite character of the single-particle density matrix.

## 3. Non-Markovian Dissipation Models

To investigate phonon-induced electronic dissipation versus decoherence via non-Markovian approaches, we shall consider a many-body Hamiltonian of the following form:

(59) $$\hat{H}={\hat{H}}_{c}+{\hat{H}}_{p}+{\hat{H}}_{\mathrm{cp}}\;,$$

where

(60) $${\hat{H}}_{c}=\sum_{\alpha }\epsilon_{\alpha }{\hat{c}}_{\alpha }^{†}{\hat{c}}_{\alpha }$$

is the many-body version of the one-electron Hamiltonian in Equation (16),

(61) $${\hat{H}}_{p}=\sum_{q}\epsilon_{q}{\hat{b}}_{q}^{†}{\hat{b}}_{q}$$

is again the phononic Hamiltonian introduced in Section 2.2 (describing the set of noninteracting phonons of wavevector q and energy $\epsilon_{q}$), while

(62) $${\hat{H}}_{\mathrm{cp}}=\sum_{\alpha \alpha^{\prime },q}\left(g_{\alpha \alpha^{\prime }}^{q,-}{\hat{c}}_{\alpha }^{†}{\hat{b}}_{q}{\hat{c}}_{\alpha^{\prime }}+g_{\alpha \alpha^{\prime }}^{q,+}{\hat{c}}_{\alpha^{\prime }}^{†}{\hat{b}}_{q}^{†}{\hat{c}}_{\alpha }\right)$$

is the many-body version of the one-electron carrier–phonon Hamiltonian in Equation (18).

According to the general quantum-kinetic treatment first proposed in Reference [27], later reviewed in Reference [65], and then extended in Reference [44], we now introduce the set of kinetic variables. In particular, the carrier subsystem is described by the single-particle density matrix (see also Equation (33))

(63) $$\rho_{\alpha_{1}\alpha_{2}}=\langle {\hat{c}}_{\alpha_{2}}^{†}{\hat{c}}_{\alpha_{1}}\rangle \;$$

and the phonon subsystem is described by the coherent-phonon amplitude $B_{q}=\langle {\hat{b}}_{q}\rangle$ and by the phonon correlation function

(64) $$n_{q}=\langle {\hat{b}}_{q}^{†}{\hat{b}}_{q}\rangle -B_{q}^{*}B_{q}\;,$$

where $\langle \ldots \rangle =\mathrm{tr}\left\{\ldots \;\hat{\rho }\right\}$.

Starting from the usual Heisenberg equations of motion for the carrier ${\hat{c}}_{\alpha }$ and phonon ${\hat{b}}_{q}$ operators, corresponding to the total many-body Hamiltonian in Equation (59), the dynamics of the coupled carrier–phonon system is governed by the following set of coupled equations:

(65) $$\begin{array}{ccc} \frac{d\rho_{\alpha_{1}\alpha_{2}}}{dt} & = & \frac{1}{ı\hbar }\left(\epsilon_{\alpha_{1}}-\epsilon_{\alpha_{2}}\right)\rho_{\alpha_{1}\alpha_{2}}+\left[\frac{1}{ı\hbar }\sum_{\alpha_{3},q}\left(g_{\alpha_{1}\alpha_{3}}^{q,-}\varrho_{\alpha_{3}\alpha_{2}}^{q}+g_{\alpha_{3}\alpha_{1}}^{q,+}\varrho_{\alpha_{2}\alpha_{3}}^{q*}\right)\;+\;H.c.\right]\\ \frac{dB_{q}}{dt} & = & \frac{1}{ı\hbar }\left(\epsilon_{q}B_{q}+\sum_{\alpha_{1}\alpha_{2}}g_{\alpha_{1}\alpha_{2}}^{q,+}\rho_{\alpha_{1}\alpha_{2}}\right)\\ \frac{dn_{q}}{dt} & = & -\left(\frac{1}{ı\hbar }\sum_{\alpha_{1}\alpha_{2}}g_{\alpha_{1}\alpha_{2}}^{q,-}\varrho_{\alpha_{2}\alpha_{1}}^{q}\;+\;c.c.\right)+\left(\frac{1}{ı\hbar }\sum_{\alpha_{1}\alpha_{2}}g_{\alpha_{1}\alpha_{2}}^{q,-}\rho_{\alpha_{2}\alpha_{1}}B_{q}\;+\;c.c.\right)\;, \end{array}$$

where “c.c.” denotes the complex conjugate and

(66) $$\varrho_{\alpha_{1}\alpha_{2}}^{q}=\langle {\hat{c}}_{\alpha_{2}}^{†}{\hat{b}}_{q}{\hat{c}}_{\alpha_{1}}\rangle \;,$$

are the so-called phonon-assisted density matrices, accounting for the quantum-mechanical phase coherence between carriers and phonons [13,65]. Due to the presence of the latter, the above set of equations is not closed. In particular, the corresponding equation of motion

(67) $$\begin{array}{ccc} \frac{d\varrho_{\alpha_{1}\alpha_{2}}^{q}}{dt} & = & \frac{1}{ı\hbar }\left(\epsilon_{\alpha_{1}}-\epsilon_{\alpha_{2}}+\epsilon_{q}\right)\varrho_{\alpha_{1}\alpha_{2}}^{q}-\frac{1}{ı\hbar }\sum_{\alpha_{3},q^{\prime }}\left[g_{\alpha_{3}\alpha_{2}}^{q^{\prime },-}\langle {\hat{c}}_{\alpha_{3}}^{†}{\hat{c}}_{\alpha_{1}}{\hat{b}}_{q^{\prime }}{\hat{b}}_{q}\rangle +g_{\alpha_{2}\alpha_{3}}^{q^{\prime },+}\langle {\hat{c}}_{\alpha_{3}}^{†}{\hat{c}}_{\alpha_{1}}{\hat{b}}_{q^{\prime }}^{†}{\hat{b}}_{q}\rangle \right.\\ & - & \left.g_{\alpha_{1}\alpha_{3}}^{q^{\prime },-}\langle {\hat{c}}_{\alpha_{2}}^{†}{\hat{c}}_{\alpha_{3}}{\hat{b}}_{q}{\hat{b}}_{q^{\prime }}\rangle -g_{\alpha_{3}\alpha_{1}}^{q^{\prime },+}\langle {\hat{c}}_{\alpha_{2}}^{†}{\hat{c}}_{\alpha_{3}}{\hat{b}}_{q}{\hat{b}}_{q^{\prime }}^{†}\rangle \right]+\frac{1}{ı\hbar }\sum_{\alpha_{3}\alpha_{4}}g_{\alpha_{3}\alpha_{4}}^{q,+}\langle {\hat{c}}_{\alpha_{2}}^{†}{\hat{c}}_{\alpha_{4}}^{†}{\hat{c}}_{\alpha_{3}}{\hat{c}}_{\alpha_{1}}\rangle \;, \end{array}$$

brings in supplementary, higher-order, kinetic variables given by expectation values of four operators, namely carrier–phonon as well as carrier–carrier two-particle density matrices. The resulting infinite hierarchy of equations may be truncated at some level via a mean-field approximation, assuming that the role played by correlations gets less effective as the number of the involved carriers and/or phonons increases [65]. Within this scheme, the generic phonon-assisted density matrix in Equation (66) is decomposed into its mean-field factorization and a remaining correlation function:

(68) $$\varrho_{\alpha_{1}\alpha_{2}}^{q}=\rho_{\alpha_{1}\alpha_{2}}B_{q}+\delta \;\varrho_{\alpha_{1}\alpha_{2}}^{q}\;.$$

At first order, one replaces the phonon-assisted density matrix $\varrho_{\alpha_{1}\alpha_{2}}^{q}$ in Equation (65) with just its mean-field factorization $\rho_{\alpha_{1}\alpha_{2}}B_{q}$. The dynamics of the carrier subsystem then turns out to be described by a fully coherent (coh) term given by

(69) $${\left.\frac{d\rho_{\alpha_{1}\alpha_{2}}}{dt}\right|}_{\mathrm{coh}}=\frac{1}{ı\hbar }\sum_{\alpha_{3}}\left(\epsilon_{\alpha_{1}\alpha_{3}}\rho_{\alpha_{3}\alpha_{2}}-\rho_{\alpha_{1}\alpha_{3}}\epsilon_{\alpha_{3}\alpha_{2}}\right)\;,$$

where

(70) $$\epsilon_{\alpha \alpha^{\prime }}=\epsilon_{\alpha }\delta_{\alpha \alpha^{\prime }}+\left(\sum_{q}g_{\alpha \alpha^{\prime }}^{q,-}B_{q}\;+\;H.c.\right)$$

indicates the single-particle energy spectrum renormalized by off-diagonal first-order carrier–phonon contributions; in the absence of coherent phonons, $B_{q}=0$ and such renormalization vanishes. As far as the phonon subsystem is concerned, in the equation of motion for the correlation function $n_{q}$, the first-order dynamics is absent and

(71) $${\left.\frac{dn_{q}}{dt}\right|}_{\mathrm{coh}}=0\;,$$

meaning that $n_{q}$ remains fixed to its initial value, typically the equilibrium distribution $n_{q}^{{}^{\circ}}$.

To account for incoherent (dissipation versus decoherence) processes, one needs to proceed one step further in the correlation expansion. In particular, the set of kinetic variables has to be enlarged to include also the carrier–phonon correlations $\delta \;\varrho_{\alpha_{1}\alpha_{2}}^{q}$ and the related dynamics. The latter is obtained by applying a mean-field approximation to the two-particle (carrier–phonon and carrier–carrier) density-matrices in Equation (67), which are factorized in terms of all possible lowest-order kinetic variables. The resulting set of coupled kinetic equations describing both coherent (coh) and incoherent (inco) carrier–phonon contributions is

(72) $$\begin{array}{ccc} \frac{d\rho_{\alpha_{1}\alpha_{2}}}{dt} & = & {\left.\frac{d\rho_{\alpha_{1}\alpha_{2}}}{dt}\right|}_{\mathrm{coh}}+{\left.\frac{d\rho_{\alpha_{1}\alpha_{2}}}{dt}\right|}_{\mathrm{inco}}\\ \frac{d\delta \;\varrho_{\alpha_{1}\alpha_{2}}^{q}}{dt} & = & \sum_{\alpha_{3}\alpha_{4}}L_{\alpha_{1}\alpha_{2},\alpha_{3}\alpha_{4}}^{q}\delta \;\varrho_{\alpha_{3}\alpha_{4}}^{q}+\frac{1}{ı\hbar }\sum_{\alpha_{3}\alpha_{4}}g_{\alpha_{4}\alpha_{3}}^{q,+}\left(n_{q}+1\right)\rho_{\alpha_{4}\alpha_{2}}\left(\delta_{\alpha_{1}\alpha_{3}}-\rho_{\alpha_{1}\alpha_{3}}\right)\\ & - & \frac{1}{ı\hbar }\sum_{\alpha_{3}\alpha_{4}}g_{\alpha_{4}\alpha_{3}}^{q,+}n_{q}\rho_{\alpha_{1}\alpha_{3}}\left(\delta_{\alpha_{4}\alpha_{2}}-\rho_{\alpha_{4}\alpha_{2}}\right)\\ \frac{dB_{q}}{dt} & = & \frac{1}{ı\hbar }\left(\epsilon_{q}B_{q}+\sum_{\alpha_{1}\alpha_{2}}g_{\alpha_{1}\alpha_{2}}^{q,+}\rho_{\alpha_{1}\alpha_{2}}\right)\\ \frac{dn_{q}}{dt} & = & {\left.\frac{dn_{q}}{dt}\right|}_{\mathrm{inco}} \end{array}$$

where

(73) $${\left.\frac{d\rho_{\alpha_{1}\alpha_{2}}}{dt}\right|}_{\mathrm{inco}}=\frac{1}{ı\hbar }\sum_{\alpha_{3},q}\left(g_{\alpha_{1}\alpha_{3}}^{q,-}\delta \;\varrho_{\alpha_{3}\alpha_{2}}^{q}+g_{\alpha_{3}\alpha_{1}}^{q,+}\delta \;\varrho_{\alpha_{2}\alpha_{3}}^{q*}\right)+H.c.\;,$$

(74) $${\left.\frac{dn_{q}}{dt}\right|}_{\mathrm{inco}}=-\frac{1}{ı\hbar }\sum_{\alpha_{1}\alpha_{2}}g_{\alpha_{1}\alpha_{2}}^{q,-}\delta \;\varrho_{\alpha_{2}\alpha_{1}}^{q}+c.c.\;,$$

while

(75) $$L_{\alpha_{1}\alpha_{2},\alpha_{3}\alpha_{4}}^{q}\;=\;\frac{1}{ı\hbar }\left(\epsilon_{\alpha_{1}\alpha_{3}}\delta_{\alpha_{2}\alpha_{4}}\;-\;\delta_{\alpha_{1}\alpha_{3}}\epsilon_{\alpha_{4}\alpha_{2}}\;+\;\epsilon_{q}\delta_{\alpha_{1}\alpha_{3}}\delta_{\alpha_{2}\alpha_{4}}\right)$$

is the effective Liouville superoperator acting on the correlation function $\delta \;\varrho_{\alpha_{1}\alpha_{2}}^{q}$.

The dynamics of the carrier subsystem, described by the set in Equation (72) is non-Markovian, that is, nonlocal in time. Indeed, by formally integrating the equation of motion for the phonon-assisted correlation function, $\delta \;\varrho_{\alpha_{1}\alpha_{2}}^{q}$, and inserting the result into the equation of motion for the electronic density matrix, $\rho_{\alpha_{1}\alpha_{2}}$, one gets an integrodifferential equation showing that the time-derivative of the latter at a given time depends on its values at all previous times [65].

The third equation in Equation (72) describes the coherent-phonon dynamics and explicitly shows that, in general, the carrier–phonon coupling itself may generate a finite coherent-phonon amplitude $B_{q}$ even when the latter is initially null. The excitation of coherent phonons in semiconductors, first observed in the surface field of n-doped GaAs by optical excitation [66], is nowadays a well-established phenomenon and can be realized in systems with sufficiently low symmetry via a properly tailored ultrafast optical excitation.

Finally, the fourth equation in Equation (72), containing the incoherent term in Equation (74), is often referred to as the hot-phonon equation, since it describes how the phonon distribution $n_{q}$ may be driven out of its thermal-equilibrium value $n_{q}^{{}^{\circ}}$ via the electron–phonon coupling. Such hot-phonon effects are known to play a key role in specifically designed and optimized quantum-cascade devices [67,68,69].

Applying to the coupled set of kinetic equations in Equation (72) of the alternative Markov treatment recalled in Section 2.2 and Section 2.3, the phonon-assisted correlation $\delta \;\varrho_{\alpha_{1}\alpha_{2}}^{q}$ can be adiabatically eliminated, allowing for the derivation of the (positive-definite) nonlinear density-matrix equation in Equation (39) equipped with the carrier–phonon scattering rates in Equation (28).

In the low-density limit, Pauli factors can be neglected ($\delta_{\alpha \alpha^{\prime }}-\rho_{\alpha \alpha^{\prime }}\to \delta_{\alpha \alpha^{\prime }}$) and hot-phonon effects are absent ($n_{q}\to n_{q}^{{}^{\circ}}$); the quantum-kinetic set in Equation (72) then assumes a much simpler form. In addition to this, an initially null phonon amplitude ($B_{q}\left(0\right)=0$) will remain zero at any later time, implying that all first-order carrier–phonon energy renormalizations in Equation (70) vanish ($\epsilon_{\alpha \alpha^{\prime }}\to \epsilon_{\alpha }\delta_{\alpha \alpha^{\prime }}$) and that the electron–phonon correlation function coincides with the phonon-assisted density matrix ($\delta \;\varrho_{\alpha_{1}\alpha_{2}}^{q}\to \varrho_{\alpha_{1}\alpha_{2}}^{q}$). Under these simplifying low-density conditions, the full set of quantum-kinetic equations in Equation (72) reduces to

(76) $$\begin{array}{ccc} \frac{d\rho_{\alpha_{1}\alpha_{2}}}{dt} & = & \frac{1}{ı\hbar }\left(\epsilon_{\alpha_{1}}-\epsilon_{\alpha_{2}}\right)\rho_{\alpha_{1}\alpha_{2}}+\left[\frac{1}{ı\hbar }\sum_{\alpha_{3},q}\left(g_{\alpha_{1}\alpha_{3}}^{q,-}\varrho_{\alpha_{3}\alpha_{2}}^{q}+g_{\alpha_{3}\alpha_{1}}^{q,+}\varrho_{\alpha_{2}\alpha_{3}}^{q*}\right)\;+\;H.c.\right]\\ \frac{d\varrho_{\alpha_{1}\alpha_{2}}^{q}}{dt} & = & \frac{1}{ı\hbar }\left(\epsilon_{\alpha_{1}}-\epsilon_{\alpha_{2}}+\epsilon_{q}\right)\varrho_{\alpha_{1}\alpha_{2}}^{q}+\frac{1}{ı\hbar }\sum_{\alpha_{3}}\;\left[g_{\alpha_{3}\alpha_{1}}^{q,+}\;\left(n_{q}^{{}^{\circ}}\;+\;1\right)\rho_{\alpha_{3}\alpha_{2}}\;-g_{\alpha_{2}\alpha_{3}}^{q,+}n_{q}^{{}^{\circ}}\rho_{\alpha_{1}\alpha_{3}}\right]\;. \end{array}$$

Applying again the alternative adiabatic-decoupling scheme in Section 2.2 to the low-density coupled set in Equation (76), the latter reduces to the linear scattering superoperator in Equation (19) equipped again with the Lindblad-type carrier–phonon rates in Equation (28).

### 3.1. Low-Density Analysis

To focus on the comparison between the above recalled quantum-kinetic treatments and their Markovian counterparts, let us consider again the two-level system previously introduced (see Equations (1) and (23)). More specifically, for the simulated experiments presented in this section, the initial condition of the electronic system is still a low-density Bell state, namely $f_{a}\left(0\right)=f_{b}\left(0\right)=p\left(0\right)\ll 1$, with null coherent-phonon amplitudes, $B_{q}\left(0\right)=0$, and phonon-assisted density matrices, $\varrho_{\alpha_{1}\alpha_{2}}^{q}\left(0\right)=0$.

**Figure 6:**
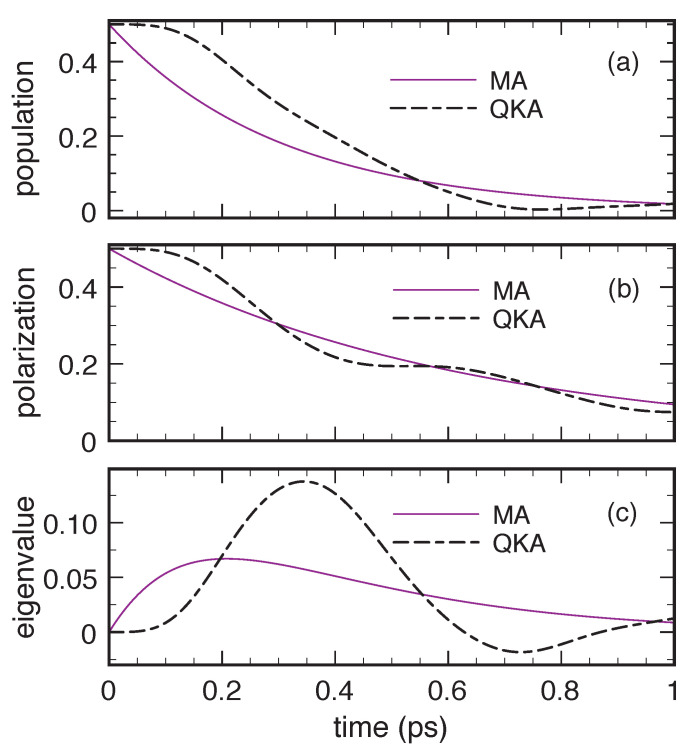
Energy dissipation and decoherence for an electronic two-level system ($\Delta_{c}=4$ meV) coupled to an acoustic-like phonon mode ($\Delta_{\mathrm{cp}}≃2$ meV) in the low-temperature and low-density limit. Excited-level relative population $f_{b}/\left(f_{a}+f_{b}\right)$ (upper panel), relative interlevel-polarization modulus $\left|p|/(f_{a}+f_{b}\right)$ (middle panel), and relative eigenvalue $\Lambda_{-}/\left(f_{a}+f_{b}\right)$ (lower panel) as a function of time obtained via the low-density quantum-kinetic approach (QKA) in Equation (76) as well as via the Markovian approach (MA) based on the Lindblad-type scattering rates in Equation (28). Reprinted from Reference [64].

We start analysing energy dissipation and decoherence effects due to the very same acoustic-like phonon mode considered in Section 2. Figure 6 compares the results of the Lindblad-type Markovian approach (MA) and the quantum-kinetic approach (QKA) for the same cases considered in Figure 2 and Figure 4. Due to the relatively strong carrier–phonon coupling, the quantum-kinetic dynamics shows significant differences from the Markovian one. In particular, the initial time derivative of the QKA excited-level population (upper panel) is null, a well-known feature of non-Markovian models, and the transient dynamics is heavily influenced by energy non-conserving transitions [7]. The combination of these two aspects initially causes a reduction of energy dissipation and decoherence, which however tends to vanish at longer times. The results in the upper and middle panels of Figure 6 undoubtedly show the effectiveness of the QKA in describing a zero-dimensional electronic system strongly coupled to acoustic phonon modes; a typical example, in this respect, is the phase-coherence versus dissipation dynamics in quantum-dot-based nanodevices [3,7]. However, in spite of these facts, positivity violations—albeit rather small—show up when looking at the eigenvalue behaviour (lower panel).

**Figure 7:**
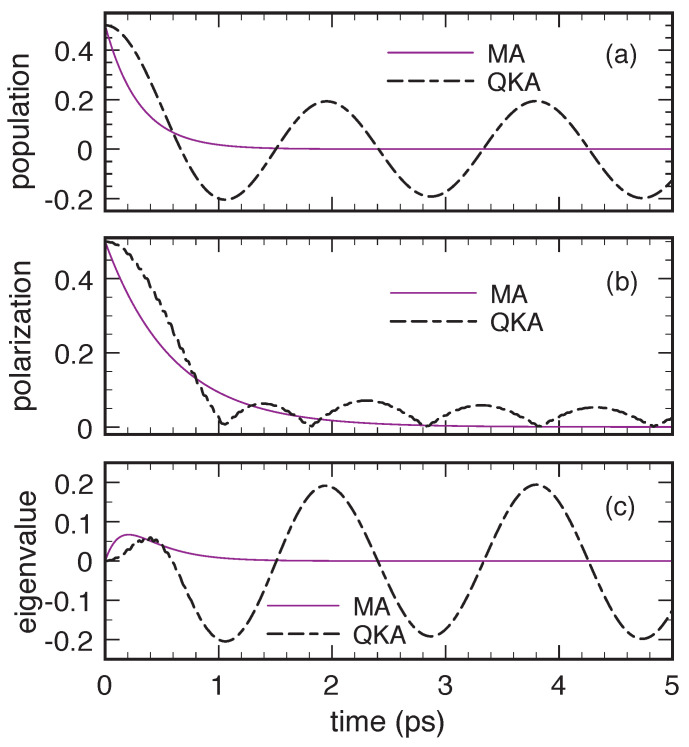
Same as in Figure 6 but for a two-level system with increased energy splitting ($\Delta_{c}=40$ meV) coupled to an optical-like phonon mode ($\Delta_{p}=4$ meV and $\Delta_{\mathrm{cp}}≃2$ meV) (see text). Reprinted from Reference [64].

We now move to the analysis of energy dissipation and decoherence effects when optical-like phonon modes are considered. Indeed, in the case of a purely zero-dimensional electronic system coupled to a dispersionless phonon mode (that is, a discrete electron-plus-phonon energy spectrum), the Markov limit is inapplicable and more refined treatments, e.g., based on the polaronic picture [70,71], are necessary. For this reason, we here assume a finite phonon bandwidth $\Delta_{p}$, centered around the electronic interlevel excitation and much smaller than the interlevel splitting $\Delta_{c}$, so that the Markov approach can be still well defined. In particular, in order to mimic electron-optical phonon scattering in GaAs-based nanostructures, we shall employ the following system parameters: $\Delta_{c}=40$ meV, $\Delta_{p}=4$ meV and a semiclassical scattering time $P_{b\to a}^{-1}=0.3$ ps, corresponding to an effective interlevel coupling energy $\Delta_{\mathrm{cp}}$ of about 2 meV. The low-density and low-temperature results for this prototypical system are reported in Figure 7, again comparing the Markovian and the non-Markovian schemes. In spite of the weak-coupling regime ($\eta =\Delta_{\mathrm{cp}}/\Delta_{c}≃0.05$), the QKA dynamics now differs significantly from the MA counterpart. Indeed, the former displays an almost dissipation-free oscillatory behaviour for the excited-level population and the interlevel polarization, while the latter has the typical exponential decay (upper and middle panels). A more relevant difference, however, is the fact that in the QKA evolution the excited-level population turns negative; this fact matches with the positivity violation in the eigenvalue analysis, reported in the lower panel.

**Figure 8:**
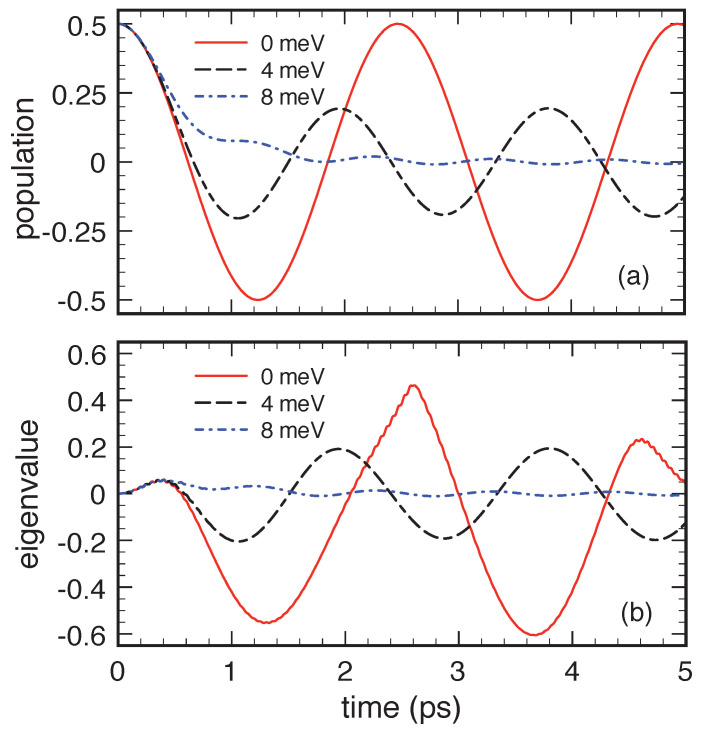
Quantum-kinetic dynamics of the excited-level relative population $f_{b}/\left(f_{a}+f_{b}\right)$ (upper panel) and of the relative eigenvalue $\Lambda_{-}/\left(f_{a}+f_{b}\right)$ (lower panel) corresponding to the same system considered in Figure 7 for different values of the optical-phonon bandwidth: $\Delta_{p}=0$ meV, 4 meV, and 8 meV. Reprinted from Reference [64].

Indeed, in this respect, the relatively small value of the optical-like phonon bandwidth is a critical issue. To investigate this aspect in a deeper way, Figure 8 compares the present QKA population and eigenvalue dynamics (dashed line in the upper and lower panel, respectively), corresponding to $\Delta_{p}=4$ meV, with the results obtained for two different values of the phonon bandwidth. In particular, $\Delta_{p}$ is set to 0 meV (solid line) and 8 meV (dash-dotted line). While, for null $\Delta_{p}$, one gets a cosine-like behavior of the population and the eigenvalue, on increasing $\Delta_{p}$, the negative regions in both the corresponding profiles are significantly reduced and the dissipation effects increased. The quantum-kinetic dynamics displayed in Figure 7 and Figure 8 claim some remarks, mainly on its oscillatory character and positivity violations.

The oscillatory behavior is clear signature of a dissipation-free dynamics; this is a well known effect [28,72] and not an artifact of the QKA: a dispersionless phonon mode, $\Delta_{p}\to 0$, does not induce any dissipation and decoherence in the electronic subsystem. Within the QKA, electronic dissipation versus decoherence phenomena result from a complex interference process involving all different electron and phonon energies. In the weak-coupling limit, all phonon-assisted density-matrix elements are expected to rotate with different frequencies: $\varrho_{\alpha_{1}\alpha_{2}}^{q}\propto \exp\left[-ı\left(\epsilon_{\alpha_{1}}-\epsilon_{\alpha_{2}}+\epsilon_{q}\right)/\hbar \right]$; this implies that the dynamics of the single-particle density matrix $\rho_{\alpha_{1}\alpha_{2}}$ (first equation in Equation (72)) is the outcome of a purely coherent and reversible interference process involving all phonon-assisted correlations. This scenario is analogous to the ultrashort temporal decay of the total polarization in a photoexcited semiconductor [73,74] resulting from the coherent superposition of microscopic polarizations rotating with different frequencies (inhomogeneous broadening), which is present also in the absence of genuine decoherence processes (homogeneous broadening). However, in the case of a zero-dimensional electronic system, the efficacy of the abovementioned interference process requires the presence of a continuum of phonon-assisted density-matrix energies much larger than the typical interstate energy splitting $\epsilon_{\alpha }-\epsilon_{\alpha^{\prime }}$. This requirement is always fulfilled in the case of the acoustic-phonon mode (see Figure 6). However, the same does not apply to the case of the optical-phonon mode and this is the main origin of the almost dissipation-free results in Figure 7 and Figure 8. It is crucial to stress once again that the anomalous dissipation dynamics pointed out so far is ascribed to the dispersionless-phonon limit only and is by no means related to the weak- versus strong-coupling regime; indeed, all the optical-phonon results presented so far (see Figure 7 and Figure 8) refer to the weak-coupling regime (η≃0.05).

The presence of unambiguous positivity violation in the QKA results in Figure 7 and Figure 8 represents a severe problem since it arises in the dispersionless regime, $\Delta_{p}\to 0$, where quantum-kinetic approaches are typically invoked as a reliable alternative to—inapplicable—Markov treatments. Therefore, while on the one hand quantum-kinetic approaches have unquestionably proven to explain a variety of ultrafast optical phenomena (like the phonon quantum beats observed, e.g., in Reference [75]) where the key role of uncompleted collisions could not be accounted for within a Markov treatment, on the other hand, the present analysis points out that caution should be used in considering QKA results as faultless overall.

To address this point more extensively, let us start noticing that, in the dispersionless limit ($\Delta_{p}\to 0$), each phonon q is characterized by the very same energy, i.e., $\epsilon_{q}\to \epsilon_{b}-\epsilon_{a}$. The effect of the whole phonon system can be therefore described via a single phonon $\bar{q}$ resonantly coupled to the two-level system via an effective coupling constant $\bar{g}$. Writing the phonon-assisted density matrix in the following form

(77) $$\left(\begin{array}{cc} \varrho_{bb}^{\bar{q}} & \varrho_{ba}^{\bar{q}}\\ \varrho_{ab}^{\bar{q}} & \varrho_{aa}^{\bar{q}} \end{array}\right)=\left(\begin{array}{cc} F_{b} & P_{ba}\\ P_{ab} & F_{a} \end{array}\right)$$

and adopting the usual rotating-wave approximation, the original quantum-kinetic equations in Equation (76) in the low-temperature limit ($n_{\bar{q}}^{{}^{\circ}}=0$) allow us to derive a set of coupled equations for $f_{b}$ and $P_{ab}$,

(78) $$\begin{array}{ccc} \frac{df_{b}}{dt} & = & 2\bar{\omega }ℑ\left(P_{ab}\right)\\ \frac{dP_{ab}}{dt} & = & -ı\bar{\omega }f_{b}\;, \end{array}$$

as well as for *p*, $F_{a}$, and $F_{b}$,

(79) $$\begin{array}{ccc} \frac{dp}{dt} & = & -ı\omega_{{}^{\circ}}p-ı\bar{\omega }\left(F_{a}-F_{b}\right)\\ \frac{dF_{a}}{dt} & = & -ı\omega_{{}^{\circ}}F_{a}-ı\bar{\omega }p\\ \frac{dF_{b}}{dt} & = & -ı\omega_{{}^{\circ}}F_{b}\;, \end{array}$$

where $\omega_{{}^{\circ}}=\Delta_{c}/\hbar$ and $\bar{\omega }=\bar{g}/\hbar$. The two sets in Equations (78) and (79) are independent and can be solved analytically; in particular, assuming an initially null phonon-assisted density matrix in Equation (77), the excited-level population evolves in time as

(80) $$f_{b}\left(t\right)=f_{b}\left(0\right)\cos\left(\sqrt{2}\;\bar{\omega }t\right)\;.$$

Such a cosine-like dynamics is in full agreement with the dispersionless-limit behavior depicted in Figure 8, confirming once again the potential limitations of the quantum-kinetic treatment (76) in describing a two-level system coupled to a single phonon $\bar{q}$.

As a further step, we shall consider the dynamics of the global (electron-plus-one phonon) system introduced above. In particular, the time evolution of its density matrix operator $\hat{\rho }$ is dictated by the following Liouville–von Neumann equation, corresponding to the total many-body Hamiltonian in Equation (59):

(81) $$\frac{d\hat{\rho }}{dt}=\frac{1}{ı\hbar }\;\left[\hat{H},\hat{\rho }\right]\;.$$

**Figure 9:**
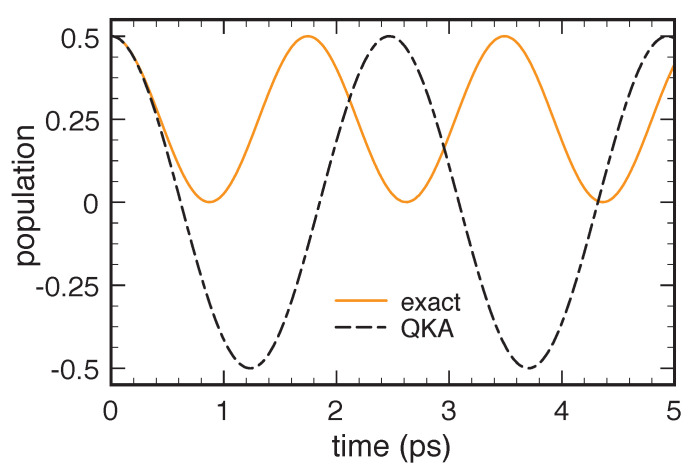
Time evolution of the excited-level relative population $f_{b}/\left(f_{a}+f_{b}\right)$ in the dispersionless case $\Delta_{p}=0$: comparison between the quantum-kinetic approach and the exact numerical approach based on the global density-matrix equation in Equation (81). Reprinted from Reference [64].

Figure 9 compares the dynamics of the excited-level population resulting from a numerical solution of the exact global density-matrix equation in Equation (81) and the quantum-kinetic outcome obtained in the case $\Delta_{p}=0$ meV. Both curves show a fully coherent dynamics and the initial zero-derivative behavior, typical of any genuine quantum-mechanical treatment. However, the QKA oscillation period comes out to be a factor of $\sqrt{2}$ larger than the exact-model one and, more importantly, while the exact result is always positive-definite, its quantum-kinetic counterpart is not.

**Figure 10:**
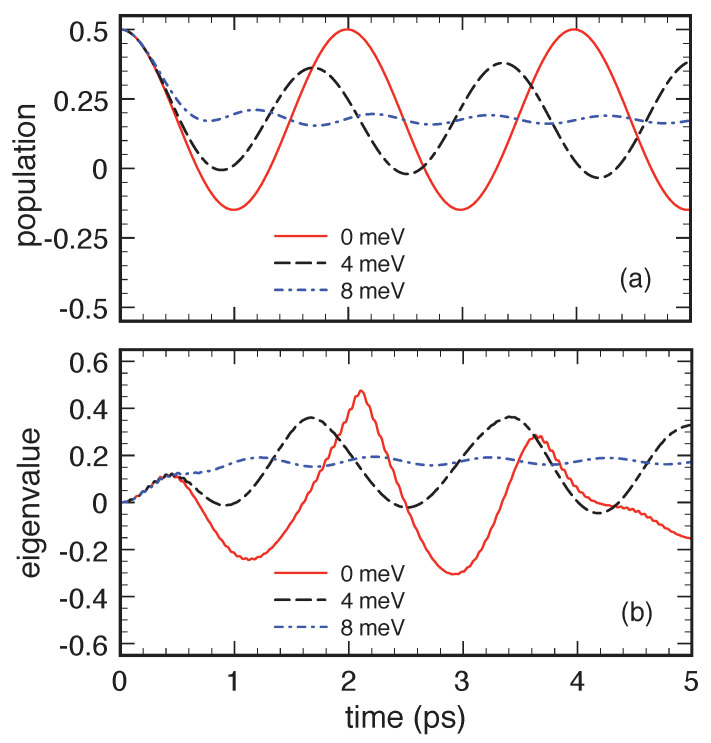
Same as in Figure 8 but for a room-temperature phonon bath (see text). Reprinted from Reference [64].

As final step of our low-density analysis, we shall now verify the possible role played by the phonon-bath temperature, which has so far been assumed equal to 0 K. To answer this question, we have therefore repeated the set of simulated experiments in Figure 8 at, e.g., room-temperature. The results are reported in Figure 10 and show a significant suppression of the negative-value regions; nevertheless, also at room temperature, in the dispersionless limit, we deal once again with a dissipation-free cosine-like population dynamics. Its oscillations are faster than the zero-temperature ones and are no more symmetric. Indeed, it is straightforward to show that the finite-temperature generalization of the analytical (zero-temperature) result in Equation (80) is given by

(82) $$f_{b}\left(t\right)=\tilde{f}+\left(f_{b}\left(0\right)-\tilde{f}\right)\cos\left(\sqrt{2}\;\tilde{\omega }t\right)$$

where

(83) $$\tilde{f}=\left(f_{a}\left(0\right)+f_{b}\left(0\right)\right)\;\frac{{\bar{n}}^{{}^{\circ}}}{2{\bar{n}}^{{}^{\circ}}+1}$$

and $\tilde{\omega }=\sqrt{2{\bar{n}}^{{}^{\circ}}+1}\;\bar{\omega }$, where ${\bar{n}}^{{}^{\circ}}\equiv n_{\bar{q}}^{{}^{\circ}}$ denotes the thermal occupation number of the dispersionless phonon mode resonantly coupled to our two-level system.

### 3.2. High-Density Analysis

The goal of this final section is to show if and to which extent the positivity violations encountered in the previous low-density analysis are present at high carrier concentrations as well.

In genuine zero-dimensional systems like, e.g., semiconductor macroatoms [3,7], the presence of a significant quantum-mechanical carrier confinement [76] determines a relevant interlevel energy splitting $\Delta_{c}$ between the two lowest electronic states. In equilibrium or quasiequilibrium conditions, the carrier occupation of all the higher-energy states is therefore negligible, so that the latter may be safely neglected and the electro-optical response of the quantum device may be properly described via our prototypical two-level system. However, opposite to the low-density regime of Section 3.1, in the high-density case, the impact of electronic degeneracy is expected to play an important role. For such a class of quantum systems and related devices, it is then vital to account for Pauli-blocking effects; to this end, the low-density quantum-kinetic set in Equation (76) should be replaced by the original (nonlinear) set in Equation (72).

**Figure 11:**
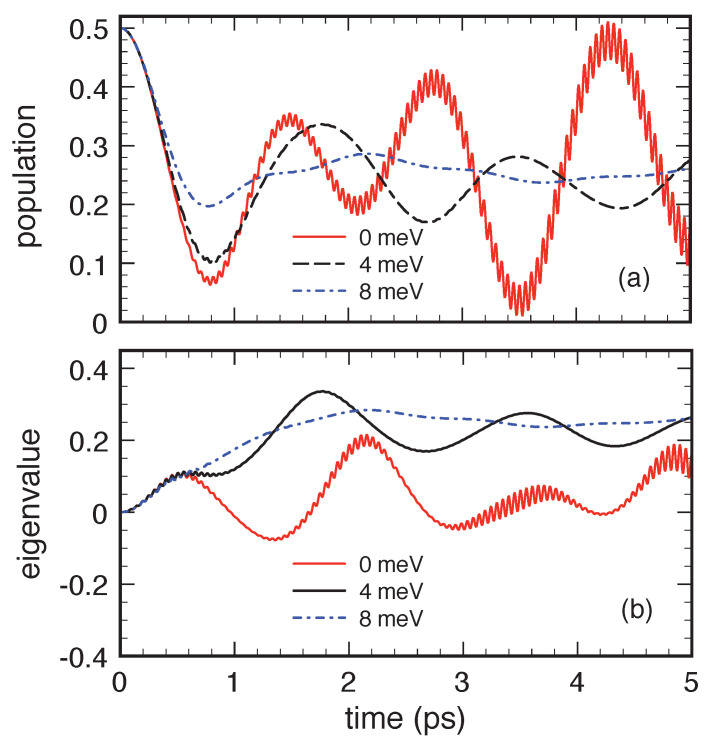
Same as in Figure 8 but including nonlinear effects via the quantum-kinetic set in Equation (72) and choosing as initial condition the (high-density) maximally degenerate Bell state $f_{a}\left(0\right)=f_{b}\left(0\right)=p\left(0\right)=\frac{1}{2}$ (see text). Reprinted from Reference [64].

To investigate the impact of electronic degeneracy, we have repeated the low-temperature simulated experiments of Figure 8, replacing the initial low-density Bell state with the maximally-degenerate Bell state $f_{a}\left(0\right)=f_{b}\left(0\right)=p\left(0\right)=\frac{1}{2}$. The results for the excited-level population and eigenvalue dynamics are reported in Figure 11 and clearly show that nonlinear effects have a strong impact on the system time evolution; indeed, comparing the high-density (or maximally-degenerate) results in Figure 11 to the low-density (or non-degenerate) results in Figure 8, we see that the inclusion of the Pauli factors $\left(\delta_{\alpha \alpha^{\prime }}-\rho_{\alpha \alpha^{\prime }}\right)$ in Equation (72) leads to a suppression of the positivity-violation signatures in the population dynamics. This is a clear indication that, in such a maximally-degenerate regime, the initial-condition parameter space leading to positivity violations is significantly reduced compared to its low-density counterpart.

## 4. Summary and Conclusions

The aim of this paper was to provide a cohesive review of the most important dissipation/ decoherence models routinely employed for the design and microscopic simulation of solid-state quantum devices, pointing out reciprocal virtues versus limitations. In particular, we focused on a few critical issues related to conventional Markov models [21] as well as to quantum-kinetic treatments [64], linking them within a common framework. More specifically, thanks to properly designed simulated experiments of a prototypical quantum-dot nanostructure (described via an electronic two-level system coupled to a phonon bath), we reached the following conclusions.

As far as Markov dissipation models are concerned, we have first shown that conventional (i.e., non-Lindblad) adiabatic-decoupling schemes may lead to positivity violations, pointing out regimes where such unphysical behaviours are particularly severe; we have then shown that these limitations may be definitely avoided adopting an alternative (i.e., Lindblad-type) adiabatic-decoupling scheme; the latter has been finally generalized to the nonlinear (i.e., degenerate) regime.

As far as dissipation models based on quantum-kinetic treatments are concerned, we have first shown that, at low temperature and low carrier concentration, the mean-field approximation may lead again to incongruous behaviors, characterized by anomalous decoherence suppression and/or positivity violations; we have then shown that the inclusion of finite-temperature conditions and/or high-density effects leads to a significant reduction of the anomalous behaviors just recalled. It is finally vital to stress that these anomalous behaviours do not affect semiconductor nanostructures with a continuous electron-plus-phonon spectrum; the latter include quantum wells and wires (where the continuous electronic spectrum allows anyway for a proper treatment of dispersionless phonon modes) as well as zero-dimensional electronic systems (quantum dots) coupled to acoustic phonons. In view of the above, the potential limitations of non-Markovian dissipation models studied in this paper (i) are expected to have negligible consequences on previous quantum-kinetic investigations and (ii) may play a significant role in the theoretical modelling of new-generation quantum nanomaterials and nanodevices operating at low carrier density and temperature and characterized by discrete electronic as well as phononic energy spectra. This concerns, in the first instance, conventional semiconductor quantum-dot based nanodevices, particularly in the regime of strong coupling to nearly dispersionless optical modes, as is typically the case for GaN-based nanostructures [77,78].

In summary, the most important conclusion of our investigation is that dissipation-induced positivity violations are ascribed not only to the adiabatic approximation but also to the mean-field approximation; indeed, while in the case of Markov treatments (based on adiabatic as well as mean-field approximations) the problem is easily avoidable by employing Lindblad-type dissipation models, for the case of non-Markovian treatments (based on the mean-field approximation only), a general strategy is still missing.

## References

[1] Nielsen M., Chuang I. (2010). Quantum Computation and Quantum Information: 10th Anniversary Edition.

[2] Bouwmeester D., Ekert A., Zeilinger A. (2013). The Physics of Quantum Information: Quantum Cryptography, Quantum Teleportation, Quantum Computation.

[3] Rossi F. (2005). Semiconductor Macroatoms: Basic Physics and Quantum-device Applications.

[4] Benson O., Henneberger F. (2009). Semiconductor Quantum Bits.

[5] Weiss U. (2012). Quantum Dissipative Systems.

[6] Ihn T. (2010). Semiconductor Nanostructures: Quantum States and Electronic Transport.

[7] Rossi F. (2011). Theory of Semiconductor Quantum Devices: Microscopic Modeling and Simulation Strategies.

[8] Breuer H., Petruccione F. (2007). The Theory of Open Quantum Systems.

[9] Shah J. (1999). Ultrafast Spectroscopy of Semiconductors and Semiconductor Nanostructures.

[10] Davies E. (1976). Quantum Theory of Open Systems.

[11] Lindblad G. (1976). Generators of quantum dynamical semigroups. Commun. Math. Phys..

[12] Bonitz M. (1998). Quantum Kinetic Theory.

[13] Haug H., Koch S. (2004). Quantum Theory of the Optical and Electronic Properties of Semiconductors.

[14] Datta S. (2005). Quantum Transport: Atom to Transistor.

[15] Haug H., Jauho A. (2007). Quantum Kinetics in Transport and Optics of Semiconductors.

[16] Jacoboni C. (2010). Theory of Electron Transport in Semiconductors: A Pathway from Elementary Physics to Nonequilibrium Green Functions.

[17] Iotti R.C., Ciancio E., Rossi F. (2005). Quantum transport theory for semiconductor nanostructures: A density-matrix formulation. Phys. Rev. B.

[18] Spohn H. (1980). Kinetic equations from Hamiltonian dynamics: Markovian limits. Rev. Mod. Phys..

[19] Taj D., Iotti R.C., Rossi F. (2009). Microscopic modeling of energy relaxation and decoherence in quantum optoelectronic devices at the nanoscale. Eur. Phys. J. B.

[20] Dolcini F., Iotti R.C., Rossi F. (2013). Interplay between energy dissipation and reservoir-induced thermalization in nonequilibrium quantum nanodevices. Phys. Rev. B.

[21] Rosati R., Iotti R.C., Dolcini F., Rossi F. (2014). Derivation of nonlinear single-particle equations via many-body Lindblad superoperators: A density-matrix approach. Phys. Rev. B.

[22] Rosati R., Rossi F. (2014). Scattering nonlocality in quantum charge transport: Application to semiconductor nanostructures. Phys. Rev. B.

[23] Rosati R., Dolcini F., Rossi F. (2015). Electron-phonon coupling in metallic carbon nanotubes: Dispersionless electron propagation despite dissipation. Phys. Rev. B.

[24] Rosati R., Reiter D.E., Kuhn T. (2017). Lindblad approach to spatiotemporal quantum dynamics of phonon-induced carrier capture processes. Phys. Rev. B.

[25] Rosati R., Lengers F., Reiter D.E., Kuhn T. (2018). Spatial control of carrier capture in two-dimensional materials: Beyond energy selection rules. Phys. Rev. B.

[26] Tran Thoai D.B., Haug H. (1993). Band-edge quantum kinetics for coherent ultrashort-pulse spectroscopy in polar semiconductors. Phys. Rev. B.

[27] Schilp J., Kuhn T., Mahler G. (1994). Electron-phonon quantum kinetics in pulse-excited semiconductors: Memory and renormalization effects. Phys. Rev. B.

[28] Fürst C., Leitenstorfer A., Laubereau A., Zimmermann R. (1997). Quantum Kinetic Electron-Phonon Interaction in GaAs: Energy Nonconserving Scattering Events and Memory Effects. Phys. Rev. Lett..

[29] Bányai L., Vu Q.T., Mieck B., Haug H. (1998). Ultrafast Quantum Kinetics of Time-Dependent RPA-Screened Coulomb Scattering. Phys. Rev. Lett..

[30] Gartner P., Bányai L., Haug H. (1999). Two-time electron-LO-phonon quantum kinetics and the generalized Kadanoff-Baym approximation. Phys. Rev. B.

[31] Vu Q.T., Haug H., Hügel W.A., Chatterjee S., Wegener M. (2000). Signature of Electron-Plasmon Quantum Kinetics in GaAs. Phys. Rev. Lett..

[32] Hannewald K., Glutsch S., Bechstedt F. (2001). Quantum-Kinetic Theory of Hot Luminescence from Pulse-Excited Semiconductors. Phys. Rev. Lett..

[33] Schmitt O.M., Thoai D.B.T., Bányai L., Gartner P., Haug H. (2001). Bose-Einstein Condensation Quantum Kinetics for a Gas of Interacting Excitons. Phys. Rev. Lett..

[34] Axt V.M., Haase B., Neukirch U. (2001). Influence of Two-Pair Continuum Correlations Following Resonant Excitation of Excitons. Phys. Rev. Lett..

[35] Betz M., Göger G., Laubereau A., Gartner P., Bányai L., Haug H., Ortner K., Becker C.R., Leitenstorfer A. (2001). Subthreshold Carrier-LO Phonon Dynamics in Semiconductors with Intermediate Polaron Coupling: A Purely Quantum Kinetic Relaxation Channel. Phys. Rev. Lett..

[36] Mieck B., Haug H. (2002). Quantum-kinetic Langevin fluctuations for exciton Bose-Einstein condensation. Phys. Rev. B.

[37] Wolterink T., Axt V.M., Kuhn T. (2003). Role of exchange interaction in Coulomb quantum kinetics. Phys. Rev. B.

[38] Herbst M., Glanemann M., Axt V.M., Kuhn T. (2003). Electron-phonon quantum kinetics for spatially inhomogeneous excitations. Phys. Rev. B.

[39] Förstner J., Weber C., Danckwerts J., Knorr A. (2003). Phonon-Assisted Damping of Rabi Oscillations in Semiconductor Quantum Dots. Phys. Rev. Lett..

[40] Seebeck J., Nielsen T.R., Gartner P., Jahnke F. (2005). Polarons in semiconductor quantum dots and their role in the quantum kinetics of carrier relaxation. Phys. Rev. B.

[41] Butscher S., Förstner J., Waldmüller I., Knorr A. (2005). Ultrafast electron-phonon interaction of intersubband transitions: Quantum kinetics from adiabatic following to Rabi-oscillations. Phys. Rev. B.

[42] Glanemann M., Axt V.M., Kuhn T. (2005). Transport of a wave packet through nanostructures: Quantum kinetics of carrier capture processes. Phys. Rev. B.

[43] Indlekofer K.M., Knoch J., Appenzeller J. (2005). Quantum kinetic description of Coulomb effects in one-dimensional nanoscale transistors. Phys. Rev. B.

[44] Krügel A., Axt V.M., Kuhn T. (2006). Back action of nonequilibrium phonons on the optically induced dynamics in semiconductor quantum dots. Phys. Rev. B.

[45] Gartner P., Seebeck J., Jahnke F. (2006). Relaxation properties of the quantum kinetics of carrier-LO-phonon interaction in quantum wells and quantum dots. Phys. Rev. B.

[46] Vu Q.T., Haug H., Koch S.W. (2006). Relaxation and dephasing quantum kinetics for a quantum dot in an optically excited quantum well. Phys. Rev. B.

[47] Nedjalkov M., Vasileska D., Ferry D.K., Jacoboni C., Ringhofer C., Dimov I., Palankovski V. (2006). Wigner transport models of the electron-phonon kinetics in quantum wires. Phys. Rev. B.

[48] Zhou J., Cheng J.L., Wu M.W. (2007). Spin relaxation in *n*-type GaAs quantum wells from a fully microscopic approach. Phys. Rev. B.

[49] Shelykh I.A., Johne R., Solnyshkov D.D., Kavokin A.V., Gippius N.A., Malpuech G. (2007). Quantum kinetic equations for interacting bosons and their application for polariton parametric oscillators. Phys. Rev. B.

[50] Zhang P., Wu M.W. (2007). Non-Markovian hole spin kinetics in *p*-type GaAs quantum wells. Phys. Rev. B.

[51] Rozbicki E., Machnikowski P. (2008). Quantum Kinetic Theory of Phonon-Assisted Excitation Transfer in Quantum Dot Molecules. Phys. Rev. Lett..

[52] Grodecka-Grad A., Förstner J. (2010). Theory of phonon-mediated relaxation in doped quantum dot molecules. Phys. Rev. B.

[53] Aeberhard U. (2011). Quantum-kinetic theory of photocurrent generation via direct and phonon-mediated optical transitions. Phys. Rev. B.

[54] Daniels J.M., Papenkort T., Reiter D.E., Kuhn T., Axt V.M. (2011). Quantum kinetics of squeezed lattice displacement generated by phonon down conversion. Phys. Rev. B.

[55] Thurn C., Axt V.M. (2012). Quantum kinetic description of spin transfer in diluted magnetic semiconductors. Phys. Rev. B.

[56] Papenkort T., Axt V.M., Kuhn T. (2012). Optical excitation of squeezed longitudinal optical phonon states in an electrically biased quantum well. Phys. Rev. B.

[57] Haug H., Doan T.D., Tran Thoai D.B. (2014). Quantum kinetic derivation of the nonequilibrium Gross-Pitaevskii equation for nonresonant excitation of microcavity polaritons. Phys. Rev. B.

[58] Cygorek M., Axt V.M. (2014). Comparison between a quantum kinetic theory of spin transfer dynamics in Mn-doped bulk semiconductors and its Markov limit for nonzero Mn magnetization. Phys. Rev. B.

[59] Papenkort T., Axt V.M., Kuhn T. (2017). Stationary Phonon Squeezing by Optical Polaron Excitation. Phys. Rev. Lett..

[60] Ungar F., Cygorek M., Axt V.M. (2017). Quantum kinetic equations for the ultrafast spin dynamics of excitons in diluted magnetic semiconductor quantum wells after optical excitation. Phys. Rev. B.

[61] Ungar F., Cygorek M., Axt V.M. (2019). Role of excited states in the dynamics of excitons and their spins in diluted magnetic semiconductors. Phys. Rev. B.

[62] Zimmermann R., Wauer J. (1994). Non-Markovian relaxation in semiconductors: An exactly soluble model. J. Lumin..

[63] Iotti R.C., Rossi F. (2015). Electronic phase coherence vs. dissipation in solid-state quantum devices: Two approximations are better than one. EPL.

[64] Iotti R.C., Rossi F. (2017). Phonon-induced dissipation and decoherence in solid-state quantum devices: Markovian versus non-Markovian treatments. Eur. Phys. J. B.

[65] Rossi F., Kuhn T. (2002). Theory of ultrafast phenomena in photoexcited semiconductors. Rev. Mod. Phys..

[66] Cho G.C., Kütt W., Kurz H. (1990). Subpicosecond time-resolved coherent-phonon oscillations in GaAs. Phys. Rev. Lett..

[67] Iotti R.C., Rossi F., Vitiello M.S., Scamarcio G., Mahler L., Tredicucci A. (2010). Impact of nonequilibrium phonons on the electron dynamics in terahertz quantum cascade lasers. Appl. Phys. Lett..

[68] Vitiello M.S., Iotti R.C., Rossi F., Mahler L., Tredicucci A., Beere H.E., Ritchie D.A., Hu Q., Scamarcio G. (2012). Non-equilibrium longitudinal and transverse optical phonons in terahertz quantum cascade lasers. Appl. Phys. Lett..

[69] Iotti R.C., Rossi F. (2013). Coupled carrier–phonon nonequilibrium dynamics in terahertz quantum cascade lasers: A Monte Carlo analysis. New J. Phys..

[70] Verzelen O., Ferreira R., Bastard G. (2002). Excitonic Polarons in Semiconductor Quantum Dots. Phys. Rev. Lett..

[71] Grange T., Ferreira R., Bastard G. (2007). Polaron relaxation in self-assembled quantum dots: Breakdown of the semiclassical model. Phys. Rev. B.

[72] Bányai L., Thoai D.B.T., Reitsamer E., Haug H., Steinbach D., Wehner M.U., Wegener M., Marschner T., Stolz W. (1995). Exciton-LO Phonon Quantum Kinetics: Evidence of Memory Effects in Bulk GaAs. Phys. Rev. Lett..

[73] Leitenstorfer A., Lohner A., Rick K., Leisching P., Elsaesser T., Kuhn T., Rossi F., Stolz W., Ploog K. (1994). Excitonic and free-carrier polarizations of bulk GaAs studied by femtosecond coherent spectroscopy. Phys. Rev. B.

[74] Haas S., Rossi F., Kuhn T. (1996). Generalized Monte Carlo approach for the study of the coherent ultrafast carrier dynamics in photoexcited semiconductors. Phys. Rev. B.

[75] Wehner M.U., Ulm M.H., Chemla D.S., Wegener M. (1998). Coherent Control of Electron-LO-Phonon Scattering in Bulk GaAs. Phys. Rev. Lett..

[76] De Rinaldis S., D’Amico I., Rossi F. (2004). Intrinsic electric field effects on few-particle interactions in coupled GaN quantum dots. Phys. Rev. B.

[77] Krummheuer B., Axt V.M., Kuhn T., D’Amico I., Rossi F. (2005). Pure dephasing and phonon dynamics in GaAs- and GaN-based quantum dot structures: Interplay between material parameters and geometry. Phys. Rev. B.

[78] Callsen G., Pahn G.M.O., Kalinowski S., Kindel C., Settke J., Brunnmeier J., Nenstiel C., Kure T., Nippert F., Schliwa A. (2015). Analysis of the exciton-LO-phonon coupling in single wurtzite GaN quantum dots. Phys. Rev. B.

